# Co-delivery of free vancomycin and transcription factor decoy-nanostructured lipid carriers can enhance inhibition of methicillin resistant *Staphylococcus aureus* (MRSA)

**DOI:** 10.1371/journal.pone.0220684

**Published:** 2019-09-03

**Authors:** Alan Hibbitts, Ainhoa Lucía, Inés Serrano-Sevilla, Laura De Matteis, Michael McArthur, Jesús M. de la Fuente, José A. Aínsa, Fabrice Navarro

**Affiliations:** 1 University Grenoble Alpes, CEA, LETI, Technologies for Healthcare and Biology division, Microfluidic Systems and Bioengineering Lab, Grenoble, France; 2 Departamento de Microbiología, Facultad de Medicina, Universidad de Zaragoza, Zaragoza, Spain; 3 CIBER Enfermedades Respiratorias (CIBERES), Instituto de Salud Carlos III, Madrid, Spain; 4 Instituto de Nanociencia de Aragón (INA), Universidad de Zaragoza, Zaragoza, Spain; 5 CIBER Bioingeniería, Biomateriales y Nanomedicina (CIBER-BBN), Instituto de Salud Carlos III, Madrid, Spain; 6 University of East Anglia, Norwich Medical School, Norwich, United Kingdom; University of Nebraska Medical Center, UNITED STATES

## Abstract

Bacterial resistance to antibiotics is widely regarded as a major public health concern with last resort MRSA treatments like vancomycin now encountering resistant strains. TFDs (Transcription Factor Decoys) are oligonucleotide copies of the DNA-binding sites for transcription factors. They bind to and sequester the targeted transcription factor, thus inhibiting transcription of many genes. By developing TFDs with sequences aimed at inhibiting transcription factors controlling the expression of highly conserved bacterial cell wall proteins, TFDs present as a potential method for inhibiting microbial growth without encountering typical resistance mechanisms. However, the efficient protection and delivery of the TFDs inside the bacterial cells is a critical step for the success of this technology. Therefore, in our study, specific TFDs against *S*. *aureus* were complexed with two different types of nanocarriers: cationic nanostructured lipid carriers (cNLCs) and chitosan-based nanoparticles (CS-NCs). These TFD-carrier nanocomplexes were characterized for size, zeta potential and TFD complexation or loading efficiency in a variety of buffers. *In vitro* activity of the nanocomplexes was examined alone and in combination with vancomycin, first in methicillin susceptible strains of *S*. *aureus* with the lead candidate advancing to tests against MRSA cultures. Results found that both cNLCs and chitosan-based carriers were adept at complexing and protecting TFDs in a range of physiological and microbiological buffers up to 72 hours. From initial testing, chitosan-TFD particles demonstrated no visible improvements in effect when co-administered with vancomycin. However, co-delivery of cNLC-TFD with vancomycin reduced the MIC of vancomycin by over 50% in MSSA and resulted in significant decreases in viability compared with vancomycin alone in MRSA cultures. Furthermore, these TFD-loaded particles demonstrated very low levels of cytotoxicity and haemolysis *in vitro*. To our knowledge, this is the first attempt at a combined antibiotic/oligonucleotide-TFD approach to combatting MRSA and, as such, highlights a new avenue of MRSA treatment combining traditional small molecules drugs and bacterial gene inhibition.

## Introduction

Antimicrobial resistance to conventional antibiotics is an increasingly serious threat to global public health that requires urgent action. Typically, antimicrobial-resistant infections carry higher incidents of mortality and present a considerable economic burden of over 20 billion dollars per year in the US alone [1]. This translates to approximately 23,000 deaths in the US and 33,000 in the EU annually as a direct result of an antimicrobial-resistant infection [1, 2] with global mortality expected to rise to up to 300 million deaths by 2050 [1]. This is especially concerning for developing nations as recent studies have predicted a greater tendency for antimicrobial resistance in Sub-Saharan Africa and parts of South America [3].

Of the multitude of microorganisms currently presenting antimicrobial resistant strains, *Staphylococcus aureus* represents a particularly serious challenge to healthcare professionals. *S*. *aureus* is a versatile Gram-positive human pathogen that is commonly found in the respiratory tract, open wounds and the urinary tract among others [4]. Even prior to the surge in antimicrobial resistance, these infections ranged in severity from relatively mild infections of the skin and soft tissue to life-threatening sepsis such as toxic shock syndrome [4]. The emergence of strains resistant to methicillin and other antimicrobial agents has added to the cost and length of treatment resulting in growing concern among medical professionals [5]. This is especially so in the hospital environment due to the higher mortality rates arising from systemic methicillin-resistant *S*. *aureus* (MRSA) infections [6, 7]. Furthermore, the World Health Organisation (WHO) published recently that there is a high priority for developing novel antimicrobials against MRSA (https://www.who.int/news-room/detail/27-02-2017-who-publishes-list-of-bacteria-for-which-new-antibiotics-are-urgently-needed). Currently in Europe, rates of MRSA infection can be found to be as high as 24% of hospital patients or 54% of all *S*. *aureus* detected [6, 8]. Resistance to first-line drugs to treat infections caused by *S*. *aureus* is widespread. Patients infected with MRSA are estimated to be 64% more likely to die than those infected with a non-resistant form of *S*. *aureus*.

First approved in 1958 by the FDA, vancomycin is effective against Gram positive bacterial infections through inhibition of bacterial cell wall synthesis [9, 10]. Vancomycin continues to be widely used, especially to combat the rising number of MRSA infections that are now appearing. Although vancomycin has been used for over 40 years, it remains the standard treatment for infections caused by MRSA with the obvious dangers of this over-reliance now becoming apparent. Specifically, reports describing clinical failures of vancomycin treatment due to the emergence of *S*. *aureus* with reduced vancomycin susceptibility have now been published [11, 12]. Doses of vancomycin needed for treatment of MRSA are now rising and, as a result, the prospect of treatment associated side effects (particularly nephrotoxicity) must now be considered for continued successful treatment [13, 14]. In addition, the use of a high dose vancomycin treatment regime against deep-seated infections has been found to have little to no impact on MRSA eradication while still resulting in nephrotoxicity [15].

**Fig 1:**
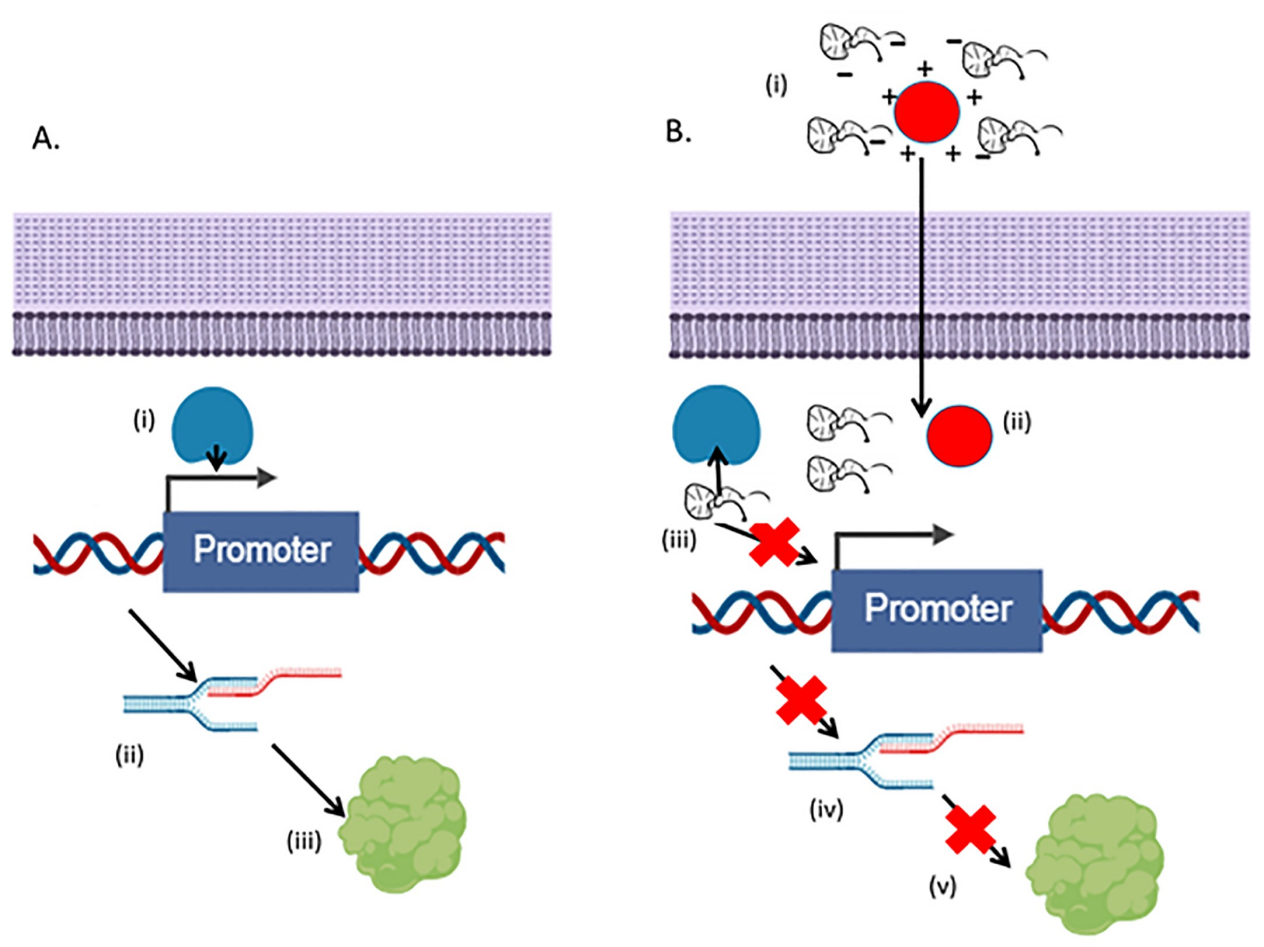
Schematic of nanoparticle-mediated TFD delivery and inhibition of *S*. *aureus* replication. A) Normal transcription process whereby (i) the transcription factor protein (s) bind their relevant promoter region which results in (ii) transcription and mRNA production which is then (iii) translated to functional protein. B) (i) Following administration of TFD-nanoparticles, (ii) these traverse the bacterial cell wall and membrane and TFD is decomplexed (iii) TFDs competitively inhibit the transcription factor binding to DNA promotor region in the bacterial chromosome and therefore (iv) considerably reduce or even inhibit transcription and (v) translation of crucial cell wall proteins (image made in BioRender—biorender.com).

To address the growing threat of antibiotic resistance, effective alternatives to the use of antibiotics are in high demand [16]. Recent research among the authors has focused on the development of transcription factor decoys (TFDs) as a new avenue of inhibiting bacterial replication. TFDs are short length oligonucleotides (10–80 base pairs) carrying the conventional binding sequence of a bacterial essential transcription factor [17]. When a bacterial cell is transformed with these molecules, the TFDs outnumber the native promoter binding sites in the chromosome; hence TFDs competitively bind to the transcription factor proteins, which will bind less efficiently to the native promoters in the bacterial genome. This in turn inhibits mRNA production, usually of multiple genes, and consequently the targeted proteins are not produced (Fig 1). This technology has previously been applied to both eukaryotic and prokaryotic organisms [18, 19] and presents a potentially new means of MRSA therapy while avoiding typical antimicrobial resistance mechanisms associated with small molecule drug treatment.

Specifically, our study has focused on the use of a TFD targeted to the WalR transcription regulator of the WalK/R two-component regulatory system of *S*. *aureus*. This protein is highly conserved and essential for the viability of *S*. *aureus*, playing a central role in controlling cell wall metabolism and membrane composition [20]. In addition, WalK/R has also been reported to be implicated in resistance to vancomycin [21] and so targeting of this transcription factor may yield additional benefits such as increasing vancomycin susceptibility of TFD-treated bacterial cells.

Like other nucleic acid-based technologies, the successful delivery and protection of the cargo prior to reaching the inside of the bacteria is an essential step in order to achieve an efficient therapeutic effect. In the case of nucleic acid delivery to Gram-positive *S*. *aureus*, it is also important to consider the cell wall which consists of a phospholipid membrane decorated with a peptidoglycan layer and is typically 20–40 nm thick. Therefore, this study investigated the suitability of two different nanoparticle systems to deliver TFDs to both methicillin susceptible *S*. *aureus* (MSSA) and MRSA. The nanocarriers investigated were developed in-house and consisted of a cationic nanostructured lipid carrier (cNLC) and chitosan-based nanocarrier (CS-NCs).

**Fig 2:**
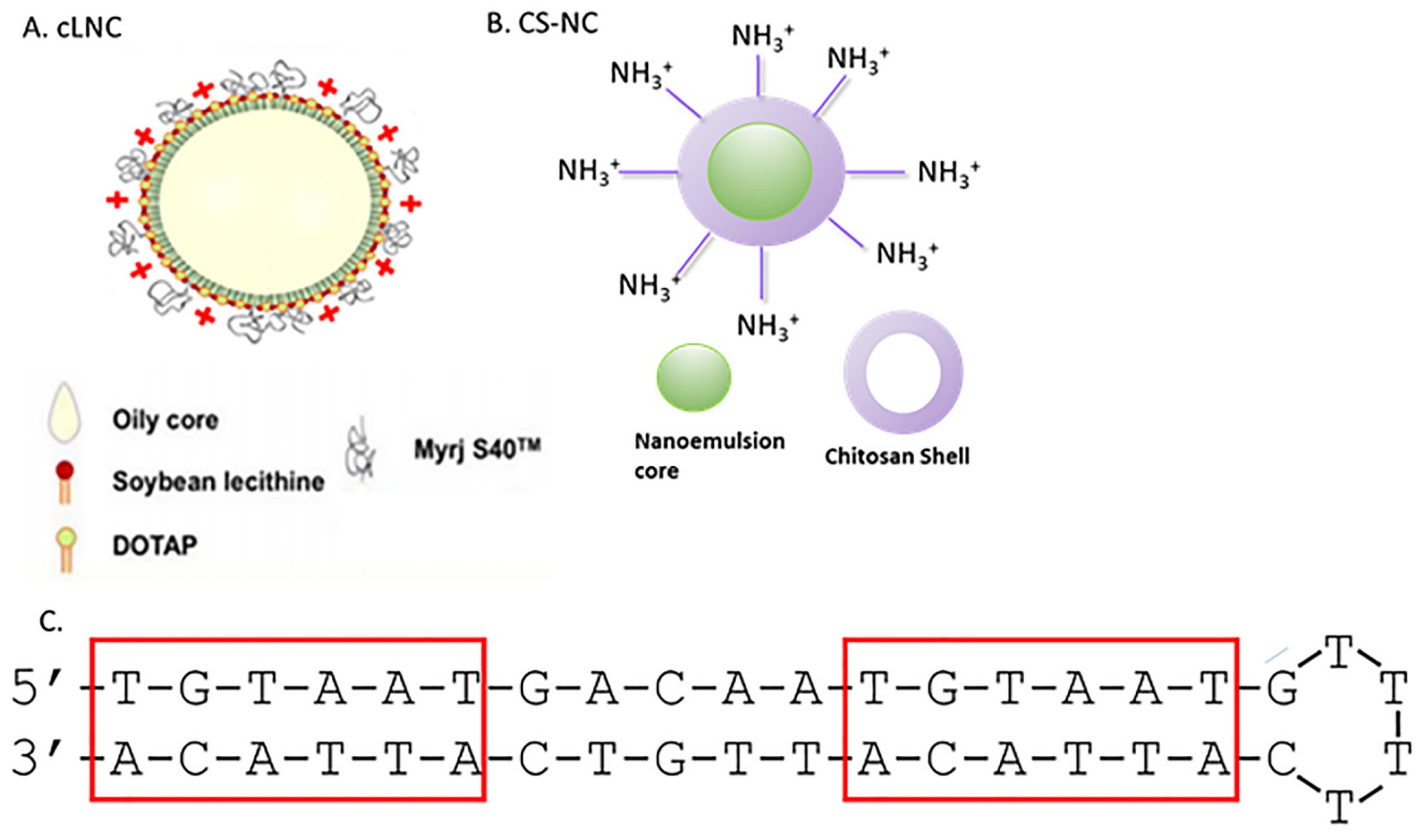
Composition of nanoparticles tested for TFD delivery. A) cationic nanostructured lipid carriers and B) chitosan based nanocarrier (CS-NCs) and C) Structure and properties of the WalR TFD (redrawn from [17, 22]).

The cNLCs consisted of an oil and wax emulsion surrounded by a PEG and cationic lipid corona (Fig 2A) and the chitosan particles consisted of an oily core with a nanogel polysaccharidic shell (Fig 2B). Both of these particle systems have previously been found to be suitable for the delivery of the small molecule antibiotic bedaquiline [22] and it was expected that the cationic nature of these systems would allow for efficient complexation of the negatively charged TFD. Following complexation, the TFD-nanoparticles were tested for stability and changes in physico-chemical characteristics following incubation in a variety of relevant buffers and advanced to an initial *in vitro* screen of antibacterial activity against a MSSA strain followed by testing against an MRSA strain for the best performing TFD-nanoparticles. Crucially, TFD-nanoparticle complexes were also tested simultaneously with vancomycin over a variety of doses with the results described herein.

## Materials and methods

### Materials

Reagents required for formulation were sourced from the same suppliers as previously described [22]. Tween 20 and absolute EtOH, were purchased from Panreac Química S.L.U (Barcelona, Spain). Span 85 (sorbitanetrioleate), oleic acid and chitosan (medium molecular weight) were purchased from Sigma-Aldrich Pte. Ltd. (Singapore). Bedaquiline was obtained from AURUM Pharmatech LLC (Franklin Park, NJ, USA). Bis(sulfosuccinimidyl) suberate (BS3) was purchased from Pierce Biotechnology Inc. (Rockford, IL, USA) and α-methoxy-ω-amino poly(ethylene glycol) (PEG-MW 5000 Dalton) from IRIS Biotech GmbH (Marktredwitz, Germany). Myrj S40 (PEG 40 stearate, 1980 Da) and Super Refined Soybean Oil were obtained from Croda Uniquema (Chocques, France). Suppocire NB was purchased from Gattefosse S.A. (Saint-Priest, France). Lipoid S75-3 (soybean lecithin at 69% of phosphatidylcholine) and hydrogenated S75 were provided by Lipoid GmbH (Ludwigshafen, Germany). All the products were pharmaceutical grade and used as received. DOTAP (1,2-dioleoyl-3-trimethylammonium-propane (chloride salt)) was purchased from Avanti Polar Lipids, Inc. (Alabaster, Alabama, USA). Water (double processed tissue culture, endotoxin-free) used in all nanocapsule synthesis was from Sigma-Aldrich.

### Transcription factor decoy synthesis

The WalR TFD was manufactured and purified by HPLC at AxoLabs (Kulmbach, Germany). It consisted of 40 base pairs consisting of the following oligonucleotides: 5’-TGTAATGACAATGTAATGTTTTCATTACATTGTCATTACA-3’ (molecular weight 12902) which contains the two TGTAAT hexamers separated by five nucleotides that are typical of WalR binding sites. Specifically, this binding site is based on the characterized WalR binding stream within the *S*. *aureus lytM* promoter [20] that is recognized by the direct repeat of two similar hexamer sequences (5’-TGTAAT-3’ and 5’-TGTATT-3’) separated by a 5 bp spacer. The nucleotides were suspended in ultrapure water to a working concentration of 1mg/ml. Similarly, a negative control TFD was fabricated in the same manner to validate the sequence specific nature of the TFD. In this case, a 30 base pair length TFD (molecular weight 9655 Da) was formed as previously described specific for the WhiB7 transcription factor binding site. This transcription factor is species specific to *Mycobacteria* [23, 24] and does not play a role in *S*. *aureus* replication or viability.

### Cationic nanostructured lipid carrier formulation

cNLCs were formulated using a specific design of experiment approach combined with nanoemulsion and sonication techniques as previously described.[22, 25] Briefly, a premix of soybean oil, Suppocire NC^™^, lecithin DOPE and DOTAP was weighed and 80 μL of a 10 mM DiD solution was added for fluorescent particles. The organic premix was then dissolved in CH_2_Cl_2_ in a 5 mL vial and the solvent was evaporated at 50 °C under a continuous stream of argon. The continuous aqueous phase, composed of MyrjTM 52 PEG and the appropriate amount of aqueous buffer (PBS), was prepared separately and added to the vial containing the evaporated organic phase. The vial was placed in a 60°C water bath and the mixture was sonicated for 10 min using a VCX750 ultrasonic processor (power output 190 W, 3-mm probe diameter, Sonics). cNLCs were purified via overnight dialysis at room temperature against 1000 times their volume in the appropriate aqueous buffer (12–14,000 Da MW cut off membranes, ZelluTrans). Finally, the cNLC dispersion was filtered through a 0.22 *μ*m Millipore membrane under aseptic conditions and stored at 4°C until required.

### Formation and physico-chemical characterization of cNLC-TFD nanocomplexes

cNLC-TFD nanocomplexes were formed using electrostatic interactions at a variety of cNLC nitrogen to TFD phosphate (N/P) ratios depending on the experimental design. To formulate nanocomplexes, the desired amount of 1 mg/ ml TFD was incubated at room temperature with its corresponding amount of cNLC for 20 minutes to allow for spontaneous nanocomplex formation. For example, for N/P = 32 and a final TFD concentration of 100 nM in 1 ml, it would be necessary to add 25.27 μl of a 2 mg/ml cNLC solution. Nanocomplexes were then diluted using the appropriate buffer to the desired final TFD concentration depending on the experiment (typically 33 nM– 1 μM).

In order to assess the effect of the various buffers used in this study on the cNLC-TFD complex stability, test complexes were formulated in phosphate buffered saline (PBS), tryptic soy broth (TSB), A549 cell culture medium (Ham′s F12K + 2mM Glutamine + 10% Foetal Bovine Serum) and human umbilical vein endothelial cell (HUVEC) culture medium (EndoGRO kit (Millipore)) and examined after 0, 24 and 72hrs for size, zeta potential and complex stability.

Dynamic light scattering was used to determine the particle hydrodynamic diameter and zeta potential analysis was undertaken using a Zeta Sizer Nano ZS (Malvern Instruments). cNLC-TFD nanocomplexes were diluted to a TFD concentration of 1 μM in their selected buffer and were then further diluted 1:50 in 10mM NaCl and transferred in Zeta Sizer Nano cells (Malvern Instrument) before each measurement. Measurements were performed in triplicate for size analysis and quadruplicate for zeta potential analysis.

cNLC-TFD nanocomplex stability in various buffers was assessed using 4% agarose gel electrophoresis. Nanocomplexes and controls were loaded as well as nanocomplexes that were also incubated with 0.2 M NaOH at a 50/50 ratio to decomplex any TFD present which will allow the now decomplexed TFD oligonucleotide to fluoresce under UV light once more. Gels were run for 20 mins and visualised under UV light for nucleic acid band formation.

### Formation and physico-chemical characterization of chitosan-TFD nanocomplexes

#### TFD entrapment in chitosan nanocarriers

The general procedure for the preparation of chitosan nanocarriers used in this work was previously reported [22] and it is based on the formation of a nanoemulsion core coated with a chitosan shell. TFDs were bound to the outer chitosan polymeric shell of the nanocarrier *via* electrostatic interaction during nanoemulsion formation as opposed to later complexation with the finished particles. The first step of nanocarrier synthesis was identical to the general procedure. Briefly, an organic solution containing 40 mg oleic acid and 8.6 mg Span 85 solved in 4 mL of absolute ethanol was added under magnetic stirring to an aqueous solution containing 13.6 mg Tween 20 in 8 mL water. The mixture was left under stirring during 15 minutes at room temperature for the formation of the nanoemulsion. At this point, 0.5 mL of a 5 mg/mL chitosan solution containing the desired amount of TFD (50, 100 or 200 **μ**g) was added to the nanoemulsion. TFD solution was prepared in water at a concentration of 1 mg/mL. The mixture was left 15 minutes more under stirring and it was finally added to 15 mL of a 50 mM Na_2_SO_4_ for the ionotropic gelation of the polymeric shell. As in the case of nanocarriers without TFD, the solid was separated by ultracentrifugation (30 minutes, 69673 G, 10°C), washed with 10 mL of water, centrifuged again and resuspended in water. The concentration of the nanocarriers in water suspension was determined by measuring the weight of the sample after freeze-drying. Sterile nanocarriers were prepared in a vertical laminar flow hood and all the reagents and materials used had been previously sterilized by UV irradiation, washing with 70% Ethanol, filtration through 0.22 μm PVDF filters (Millex syringe driven filter unit) or by dry heat treatment (180°C).

#### Determination of TFD entrapment efficiency and drug loading

The amount of entrapped TFD was determined by means of agarose gel electrophoresis (20 min at 90V with 1% agarose gels) stained with GelRed and the following analysis of fluorescence intensity of the band corresponding to TFD using a Gene Genius Syngene Transilluminator and ImageJ software. Different amounts of free TFD of a known concentration were included to obtain a calibration curve to quantify the loaded TFD. With this value, encapsulation efficiency and drug loading were calculated. Entrapment efficiency (EE) was calculated as the percentage of entrapped TFD over the amount added initially for the preparation of nanocarriers. Drug loading (DL) was expressed also as percentage and was the ratio between entrapped TFD and the total weight of chitosan nanocarrier in the formulation.

#### Characterization of chitosan nanocarriers

The characterization of the obtained material was carried out by means of Dynamic Light Scattering (DLS) analysis using a Brookhaven 90Plus DLS instrument and by means of Photo-Correlation Spectrosocopy (PCS) technique at a concentration of nanocarriers of 0.15 mg/mL. Z-potential analysis was carried out using a Plus Particle Size Analyzer (Brookhaven Instruments Corporation). In this case nanocarriers were measured at a concentration of 5 μg/mL in 1mM KCl. The stability of TFD-loaded nanocarriers toward aggregation in different media of biological interest was determined by DLS and Z-potential analysis as previously reported. TFD-loaded CS-NCs were previously incubated at 2 mg/mL and 37°C in phosphate buffered saline (PBS), BBL Trypticase Soy Broth (TSB), BBL Mueller Hinton II Broth Cation Adjusted medium (MHII) bacteria culture media and pure water for comparison. They were measured immediately after entering in contact with the medium, after 24 and 72 hours of incubation in terms of size (DLS) and surface potential.

### Bacterial strains and culture conditions

*Staphylococcus aureus* reference strains CECT-794 (Methicillin Sensitive *S*. *aureus*, ATCC 29213) and CECT 5190 (Methicillin Resistant *S*. *aureus*, ATCC 43300) were purchased from the Spanish Collection of Type Cultures (CECT) and conserved in BBL Trypticase Soy Broth in 15–17% glycerol stocks at -80°C. For each experiment, the bacteria were transferred from the frozen stock to a Columbia Agar plate with 5% Sheep Blood (BD), incubated overnight, and one colony from that plate was transferred to 5 ml of BBL Trypticase Soy Broth, then incubated 20h at 37°C and used as the pre-inoculum for the experiments.

The MIC assays were performed with BBL Mueller Hinton II Broth Cation Adjusted medium as recommended by the CLSI Standards for Antimicrobial Susceptibility Testing.

### *In vitro* activity of vancomycin and TFD nanocomplexes vs *Staphylococcus aureus*

The anti-microbial effects of vancomycin and TFD nanocomplexes were tested both separately and in combination using a 96 well plate assay as previously described by Palomino *et al*. and Ramon-García *et al*.[26, 27] The checkerboard synergy test was used to study the interaction between vancomycin and the TFD-nanocomplex against MSSA and later MRSA. Briefly, prior to the addition of bacteria, serial dilutions of vancomycin were pipetted into wells along the vertical axis and serial dilutions of TFD-nanocomplexes were pipetted into wells along the horizontal axis of a 96 well plate in parallel (S1A Fig). This allowed for the anti-microbial effect of both vancomycin and TFD nanoparticles to be tested separately and combined. Specifically, cNLC-TFD particles were tested using vancomycin concentrations ranging from 2.5 μg/ml-0.04 μg/ml against TFD concentrations of 250 nM-0.5 nM. Bacteria were added to a final concentration of 10^5^ cfu/mL, and the plates were incubated for 20 h at 37°C. At this point, bacterial inhibition was assessed using Alamar blue stain and recording a clear change in color from blue (no growth) to pink (bacterial growth) (S1B Fig). The lowest concentration that prevents a colour change was recorded as the MIC.

In the case of the MRSA cultures, bacterial growth inhibition was first assessed qualitatively for a clear change in color (due to reduction of Alamar blue) and then was also quantified using a microplate reader to determine absorbance at 570 nm and normalized to background absorbance at 600 nm. In this case, percentage of bacterial growth was calculated with respect to 100% growth in non-treated positive control bacteria. 96-well plate synergy assays were run on 3 independent occasions for cNLC-TFD particles in both MSSA (and later MRSA) cultures in order to establish vancomycin MICs and optimal TFD concentration.

Following checkerboard synergy assays, two representative concentrations of TFD were selected for further experiments with MSSA strain CECT794: 125nM and 8nM using a reduced number of samples in a 96 well plate format. This allowed for a reduced burden on TFD stocks and offered a more streamlined method of testing non-specific TFD controls These two TFD concentrations, in chitosan based CS-NC-TFDs (S2 Fig) and cNLC-TFD nanoplexes (S3 Fig), were tested in combination with serial dilutions of vancomycin, ranging from 2.5 to 0.16 μg/ml. These experiments were carried out in duplicate for a further 6 independent experiments.

### Cell viability assays

24 hours prior to testing, cells were seeded at a density of 1.25x 10^4^ cells/ well in 96 well plates. On the following day, cNLC-TFD nanocomplexes were formulated as previously described in the methods section at an N/P ratio of 32. Nanocomplexes were added at a TFD range of concentrations from 500 to33 nM in complete cell culture media to either A549 adenocarcinomic human alveolar basal epithelial cells (ATCC CCL-185) or primary Human Umbilical Vein Endothelial Cells (HUVEC, Thermofisher Scientific) and incubated at 37°C for 24 hrs. Following incubation, cells were treated with WST-1 cell viability reagent (Roche, France) according to the manufacturer’s instructions and cell viability was read using a Tecan microplate reader at 450 nm. Cell viability of treated cells was compared against those of untreated controls and cells treated with 100 μM staurosporine (Sigma-Aldrich) as a positive control and background adjusted against cell medial only wells. Viability experiments were carried out in triplicate and repeated 3 independent times.

### Haemolysis assays

Haemolysis assays were performed using whole human blood samples which were obtained from healthy donors (Etablissement Français du Sang (EFS), Grenoble, France) and were collected in citrate vacutainer tubes (Becton Dickinson, Le Pont de Claix, France). Informed consent was given by blood donors according to the ethical and legal standards of our blood supplier (EFS). Samples were used without any dilution and the blood tubes were delivered three days after withdrawal and were kept at 4 ◦C in a fridge for storage. The experiments were performed within three days after delivery.

On the day of testing, cNLC-TFD complexes were formed as previously described and diluted using the whole blood samples to a final volume of 500 μL and TFD concentrations ranging from 33–500 nM. Samples were incubated at 37°C for 90 min and then centrifuged for 15 min at 800 g. The supernatant was collected and pipetted in duplicate into a 96 well plate before being analyzed using a Tecan microplate reader at an absorbance of 540 nm. Percentage haemolysis was calculated by comparing against untreated controls. Samples were run in triplicate and tested using 6 different donors.

### Statistical analysis

Results were expressed as means ± standard error of the mean (SEM) for experiments using GraphPad Prism 5 software. Two and one-way analysis of variance (ANOVA) for differences between treatments with p-values < 0.05 considered significant, < 0.01 very significant and < 0.001 highly significant.

## Results

### Loading efficiency and stability of cNLC-TFDs nanocomplexes in biological buffers

**Fig 3:**
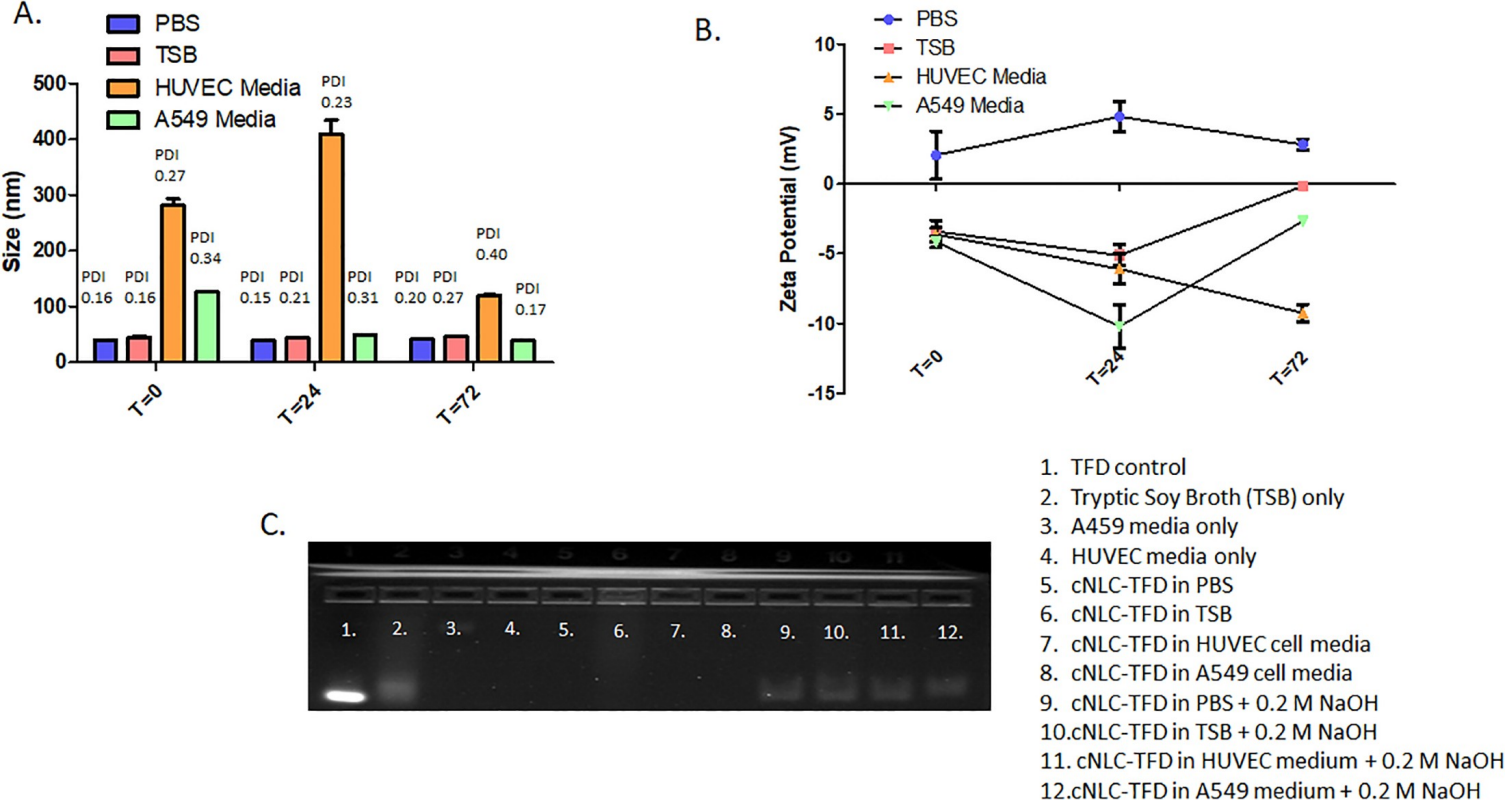
Stability analysis of cNLC-TFD nanocomplexes at N/P = 32 over a 72-hour timeframe in a variety of biological buffers. A) Size and polydispersity analysis B) zeta potential analysis C) agarose gel electrophoresis analysis of nanocomplex integrity following incubation at 37°C for 72 hours.

Following formulation of the cNLC-TFD nanocomplexes, their size, polydispersity, zeta potential and complex stability were analyzed over a 72-hour time period in a variety of biological buffers (Fig 3, S1–S3 Tables).

When analyzed for changes in nanocomplex size (Fig 3A), it was found that nanocomplexes incubated in PBS buffer and TSB culture medium exhibited high levels of colloidal stability. The size of nanocomplexes remained at approximately 45 nm throughout the 72 hours of incubation with only minor increases in PDI observed for PBS treated samples (0.16 increasing to 0.20) and a slightly more substantial increase in the case of TSB treated samples (0.16 increasing to 0.27). In contrast, nanocomplexes treated with A549 and HUVEC cells culture media demonstrated an immediate increase in size and polydispersity. This was most evident in the more complex HUVEC media, where nanocomplex size was 282 nm on the first day of analysis and increased up to a maximum size of 409 nm 24 hours later. Size increases for A549 medium-treated nanocomplexes was less pronounced with a maximum size of 129 nm recorded, however polydispersity indices for both media remained high throughout, with the highest levels of 0.4 recorded in HUVEC media-treated samples after 72 hours.

On examination of the changes in zeta potential, evidence of buffer-specific behavior was found in the results (Fig 3B). Specifically, cNLC-TFD nanocomplexes dispersed in PBS were the only samples that retained a cationic surface charge throughout the 72 hours of testing. This gave an early indication that the TFD was fully complexed by the cNLCs. In contrast, all the cell and microbiology culture media displayed roughly the same anionic surface charges (-3.6 mV to -4.1 mV) immediately after dispersion. cNLC-TFD nanocomplexes dispersed in culture media demonstrated further decreases at 24-hours incubation with the lowest zeta potential recorded by nanocomplexes dispersed in A549 medium showing -10.2 mV.

For the final aspect of cNLC-TFD characterization, the integrity of the nanocomplexes was assessed following 72-hours incubation using agarose gel electrophoresis (Fig 3C). Nancomplexes were incubated in either equal volume of dH_2_O or 0.2 M NaOH for 5 minutes before loading in the gel. Using this technique, it was demonstrated that cNLC-TFD nanocomplexes were fully complexed at N/P = 32 and maintained their integrity for up to 72 hours in each biological buffer with the TFD oligonucleotide only being released from the nanocomplex following the decomplexation in the presence of NaOH.

### Determination of TFD entrapment efficiency and drug loading in chitosan nanocarriers

Since TFD entrapment occurred during formulation of CS-NCs as opposed to after, it was necessary to assess the TFD loading prior to advancing to stability studies. The entrapped TFD was therefore quantified by agarose gel electrophoresis *via* precast gel staining with GelRed. A simple, direct method was used to quantify the TFD entrapped in the CS-NCs. Chitosan nanocarriers with the entrapped TFD were loaded into the gel at an adequate dilution without previous treatment, allowed to run to separate unentrapped TFD and then quantified against a series of samples with known amounts of the free TFD.

**Fig 4:**
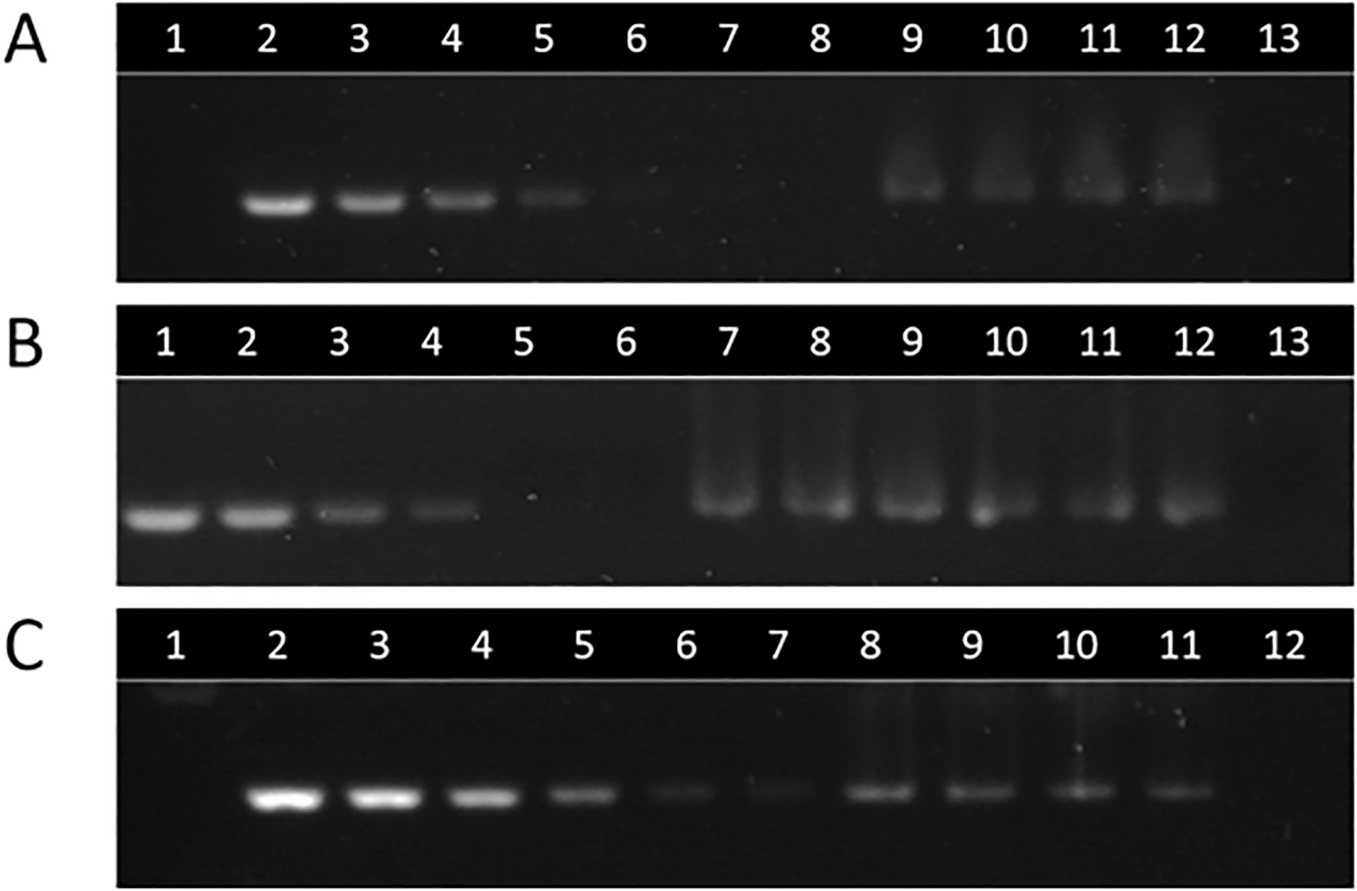
Agarose gel electrophoresis to quantify the TFD entrapped in chitosan nanocapsules. A) Addition of 50 μg TFD. Lane 1, empty CS-NCs; lanes 2–7, free TFD calibration; lane 8, empty well; lanes 9–12, TFD-CS-NCs diluted 1:2; lane 13, filtered TFD-CS-NCs. B) Addition of 100 μg TFD. Lanes 1–4, free TFD calibration; lanes 5–6, empty CS-NCs; lanes 7–9, TFD-CS-NCs diluted 1:5; lanes 10–12, TFD-CS-NCs diluted 1:10; lane 13, filtered TFD-CS-NCs. C) Addition of 200 μg TFD. Lane 1, empty CS-NCs; lanes 2–7, free TFD calibration; lanes 8–11, TFD-CS-NCs diluted 1:8; lane 12, filtered TFD-CS-NCs.

Following visualization, a band appeared in CS-NC-TFD samples at the same distance as free TFD loaded wells (Fig 4, S4 Table). This indicated that the entrapped TFD was released from the nanocarrier, most likely due to a decrease in the electrostatic forces between TFD and nanocarrier provoked by the alkaline pH of the electrophoresis buffer (pH 8.5). This was further confirmed by loading a filtered suspension of the nanocarrier in their starting aqueous medium in the gel. The absence of any band corresponding to the free TFD in this control confirmed that all the TFD molecules in the sample were associated to the nanocarrier and did not exist as free molecules in the suspension. Empty nanocarriers at the same concentration as the TFD-loaded samples were used as negative control.

Once it was established that the TFD was entrapped in the chitosan shell of the nanocarriers, the percentage entrapment efficiency and drug loading were calculated (Table 1, S5 Table). Differences in TFD loading were achieved during formulation by adding varying amounts of TFD and relating the final amount of entrapped TFD against carrier mass (as opposed to %w/w of starting materials).

**Table 1:** Drug loading and entrapment efficiency of TFD-CS-NCs.

**TFD initially added (μg)**	**50**	**100**	**200**
**Drug loading (DL) %**	0.071 ± 0.007	0.138 ± 0.016	0.190 ± 0.049
**Entrapment efficiency (EE) %**	42 ± 4	44 ± 5	24 ± 6

On analysis, it was determined that addition of 50 μg TFD resulted in a percentage drug loading (DL) of 0.071% and a percentage encapsulation efficiency (EE) of 42%. When doubling the amount of TFD to 100 μg, the DL increased (0.138%) however EE remained at 44%. However, when doubling the amount of TFD to 200 μg, the DL obtained was far from being doubled (0.190%) and the EE decreased (24%). This was understood that beyond addition of 100 μg TFD to the synthesis, the saturation point was reached and the nanocarrier was unable to entrap any further TFD. In view of these results, nanocarriers synthesized by the addition of 100 μg TFD were chosen as the best candidates for further studies, since they presented a high DL value without compromising the EE.

### Stability of TFD-loaded chitosan nanocarriers in biological buffers

Four different media have been used for the study of nanocarriers stability: water, phosphate buffer saline (PBS), tryptic soy broth (TSB) and Mueller Hinton II (MHII) culture media. MilliQ water was chosen because it is the storage medium for chitosan nanocarriers. PBS is a commonly used isotonic buffered salt solution used for intravenous injection. TSB is a general-purpose medium that is routinely used to grow most bacteria and MHII is the medium used for antibiotic susceptibility testing with methicillin-resistant strains of *S*. *aureus*. In contrast to cNLC-TFD formulations, TFD-CS-NCs were not tested in A549 and HUVEC as they were not brought forward for *in vitro* testing in mammalian culture as they were discarded due to their low levels of antimicrobial effect.

**Fig 5:**
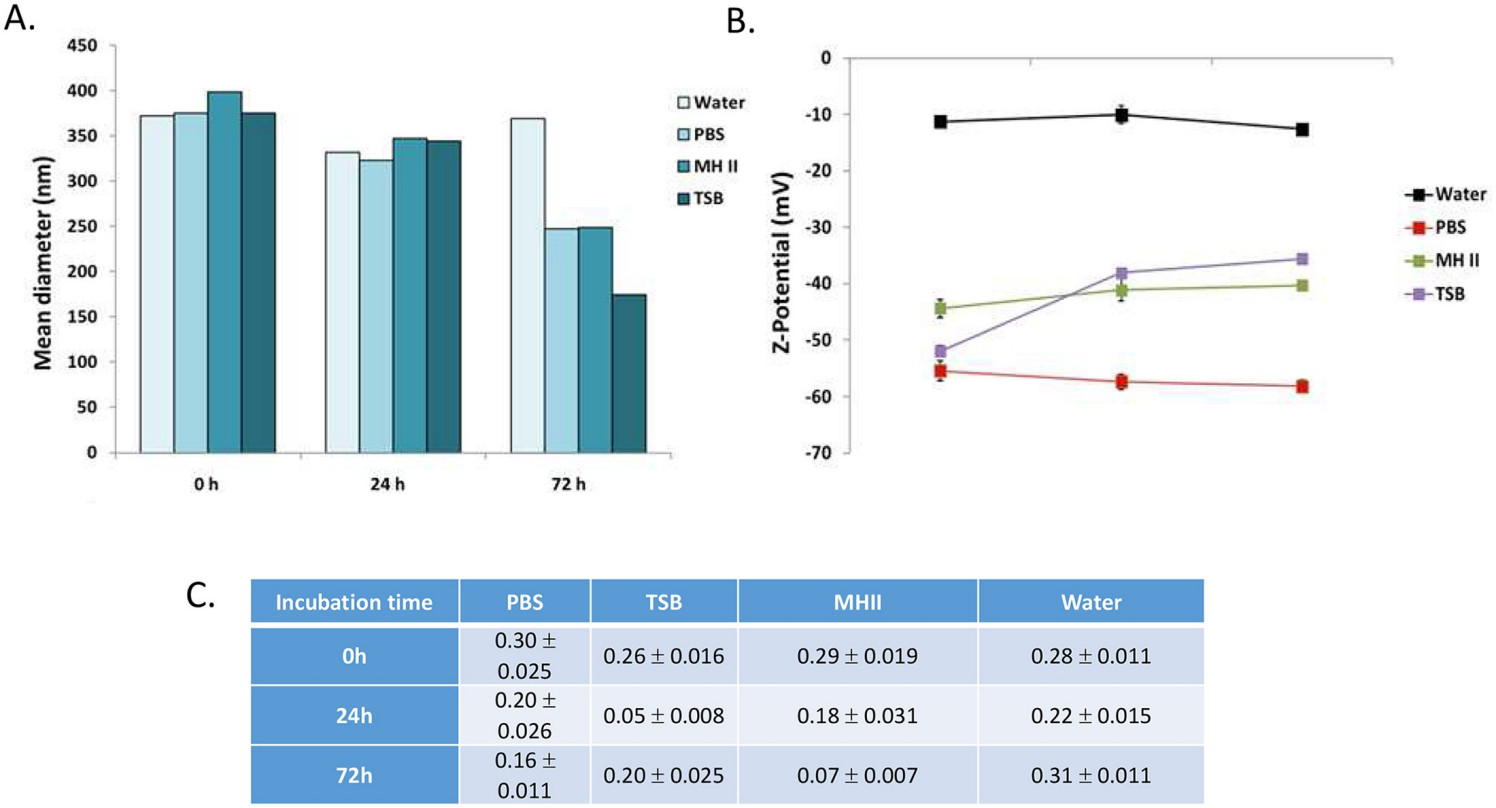
Stability analysis of TFD-CS-NC nanocarriers over a 72-hour timeframe in a variety of storage and biological buffers. A) Size analysis B) zeta potential analysis and C) polydispersity index.

When analyzed for particle size (Fig 5A, S6–S8 Tables), during the first 24 h the hydrodynamic diameter was slightly decreased independently of the buffer used for the incubation. Mean diameters were slightly smaller at 24 h than at time 0 but still comprised between 300 and 400 nm. After 72 h nanocarriers in water exhibited a similar mean diameter but nanocarriers incubated in PBS, TSB and MHII showed a pronounced decrease in their size, especially in TSB, with a mean diameter of 175 nm and with mean diameters of 250 nm in PBS and MHII. Size distribution of TFD-loaded chitosan nanocarriers is quite polydisperse in aqueous solution. A low percentage of aggregates or bigger nanoparticles was always detected but it was considered non-significant for the aim of this study.

Z-potential analysis, performed after incubation of nanocarriers in different media, evidenced some buffer-specific behavior that was attributed to a masking effect on nanocarriers surface due to the adsorption of ions and proteins contained in these media (Fig 5B). Specifically, nanocarriers dispersed in water presented a Z-potential value slightly negative (around -10 mV) that maintained constant during 72 h incubation. On the contrary, the surface potential of nanocarriers in culture media (TSB and MHII) was quite lower (between -38 and -50 mV) and even lower in PBS since in this buffered medium the value maintained almost constant around -60 mV during 72 h.

### *In vitro* activity of vancomycin in combination with TFD-nanoplexes vs *S*. *aureus* (MSSA and MRSA)

To determine the independent and combined antimicrobial abilities of vancomycin and the TFD-nanocomplexes, two strains of *S*. *aureus* were used. Initial experiments were undertaken using the MSSA reference strain CECT794, which is indicated by the CLSI guide as a standard in drug susceptibility assays. Following this, formulations displaying anti-microbial effects were brought forward for testing in CECT5190, which is the methicillin resistant strain commonly used for drug susceptibility assays.

#### TFD-nanocomplex/vancomycin synergy assays in CECT 794 MSSA

From visual assessment of the plate following Alamar blue addition, cNLC-TFD nanocomplexes did not demonstrate any antibacterial activity by themselves in concentrations up to 500 nM of TFD (S1 Fig). However, the presence of cNLC-TFD nanocomplexes in combination with vancomycin, resulted in a 50% decrease in the MIC of vancomycin, compared to the antibiotic alone from 0.6 μg/ml to 0.3 μg/ml. This was only observed when the TFD was complexed with the cNLC and this effect was clearly dependent on the concentration of TFD. When the amount of TFD was 66 nM and above, the efficacy of vancomycin was enhanced but below this concentration there was no effect evident.

Two representative concentrations of TFD-NC were selected for further experiments with CECT794: 125 nM and 8 nM. These were tested in standard growth inhibition tests with CECT 794 together with sub-MIC amounts of vancomycin. Similarly, the chitosan-based CH-NC-TFD nanocarriers were also tested in this reduced sample format. Unfortunately, the same result was not obtained for the chitosan nanocarriers, as they did not show this boosting effect for the antibiotic. (Table 2). This experiment was repeated 6 times with identical results (S2 Fig).

**Table 2:** MIC values of vancomycin and either cNLC-TFD or CH-NC-TFD nanocomplexes as well as relevant controls (n = 6) against *S*. *aureus* CECT794 strain using reduced sample assay.

	MIC vancomycin (μg/ml)					
	vanco alone	+ WalR-TFD 125nM	+ WalR-TFD 8nM	+ empty carrier	+ WalR-TFD non- encapsulated	+ non-specific (WhiB7) TFD
cNLC	0.6	0.3	0.6	0.6	0.6	0.6
CH-NC	0.6	0.6	0.6	0.6	0.6	0.6

It was also confirmed that free WalR TFD non-associated to any nanocarrier did not have any boosting effect on vancomycin (S3 Fig), reinforcing the requirement of the DNA sequence to be complexed for efficient delivery. In addition, the cNLC without any TFD associated, at a concentration equal to that present when a concentration of 125 nM TFD is achieved, does not modify the efficiency of vancomycin, thus corroborating that the observed effect is due to the oligonucleotides. Considering the lack of efficacy observed in the current guise of the chitosan-based nanocarriers for TFD delivery, it was decided to continue to MRSA studies with only the cNLC complexed TFDs.

#### TFD-nanocomplex/vancomycin synergy assays in CECT 5190 MRSA

Following initial success with the MSSA strain, the cNLC-TFD nanocomplexes were assayed to determine if the boosting effect when co-administered with vancomycin was also possible in MRSA. Vancomycin and cNLCs were tested simultaneously in a checkerboard synergy assay as demonstrated for MSSA and observed after 24 hours for changes in the MIC of vancomycin. The MRSA plates were analyzed using spectrophotometry following Alamar blue addition to establish if there were any changes in MRSA growth.

**Fig 6:**
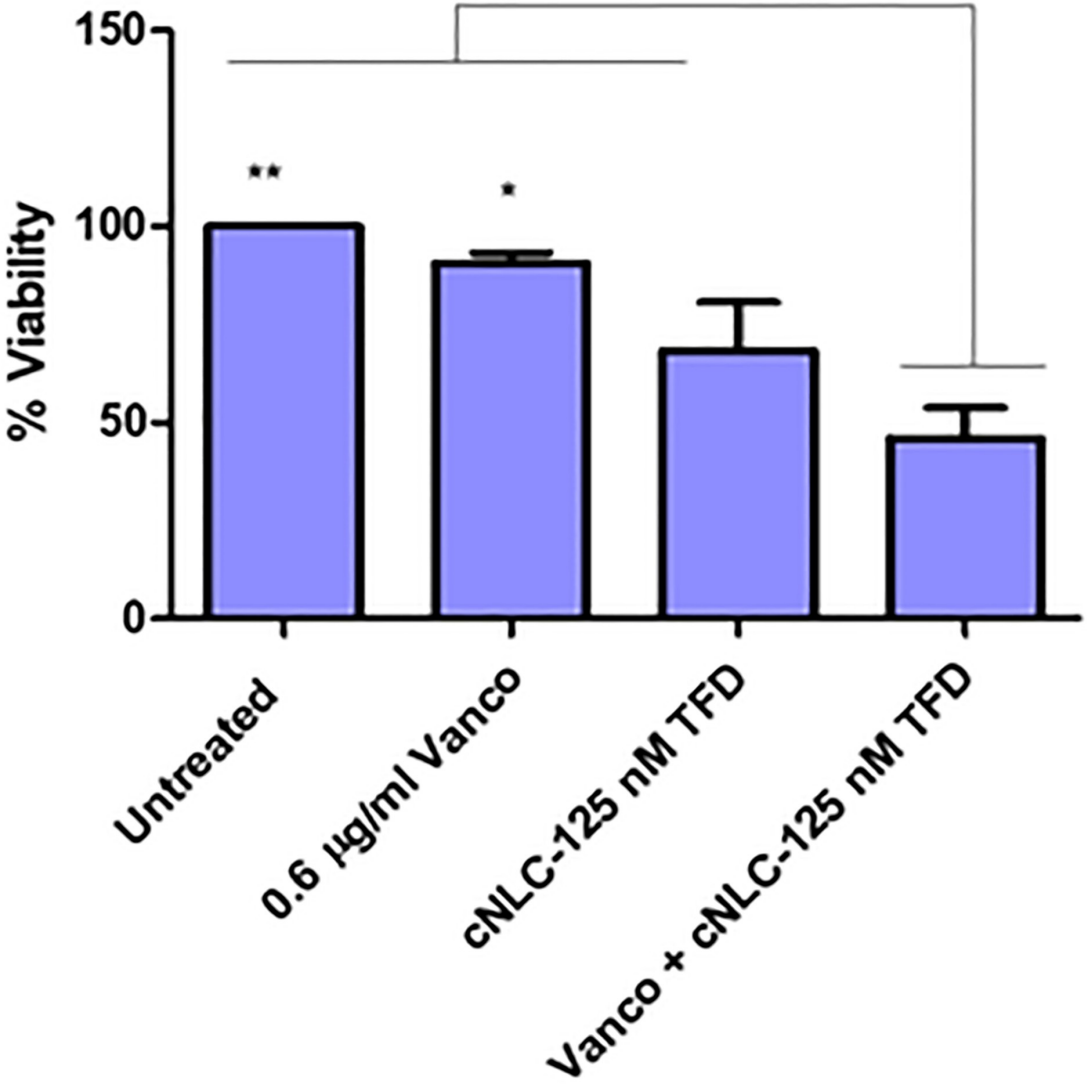
Synergy assay demonstrating enhanced antimicrobial effect against MRSA strain CECT 5190 when read at 570 nm. From left, untreated MRSA controls, MRSA treated with sub-MIC dose of 0.6 μg/ml vancomycin, MRSA treated with cNLC-TFD nanocomplexes at a TFD concentration of 125 nM and MRSA treated with dual therapy of 0.6 μg/ml free vancomycin and cNLC-125nM TFD nanocomplexes (n = 3 ±SEM, *P<0.05, **P<0.01, one-way ANOVA).

Using this method, it was found that while there was not total inhibition of the MRSA growth, there were significant decreases in viability observed when the samples were treated with cNLC-TFD nanocomplexes in the presence of vancomycin. For this strain, the MIC of vancomycin was 1.2 μg/ml and so at a concentration of 0.6 μg/ml of vancomycin alone, the bacterial growth was found to be 90% (± 4.30%) of the untreated control. In contrast, in the presence of cNLC containing 125nM of TFD, the bacterial growth for 0.6μg/ml of vancomycin was reduced to 46% (± 13.41%) with respect to untreated bacteria (Fig 6, S9 Table). This indicated that, while not completely eradicating the MRSA, the potency of the vancomycin was significantly increased when combined with cNLC-TFD nanocomplexes.

### Mammalian cell viability following administration of cNLC-TFD nanocomplexes

**Fig 7:**
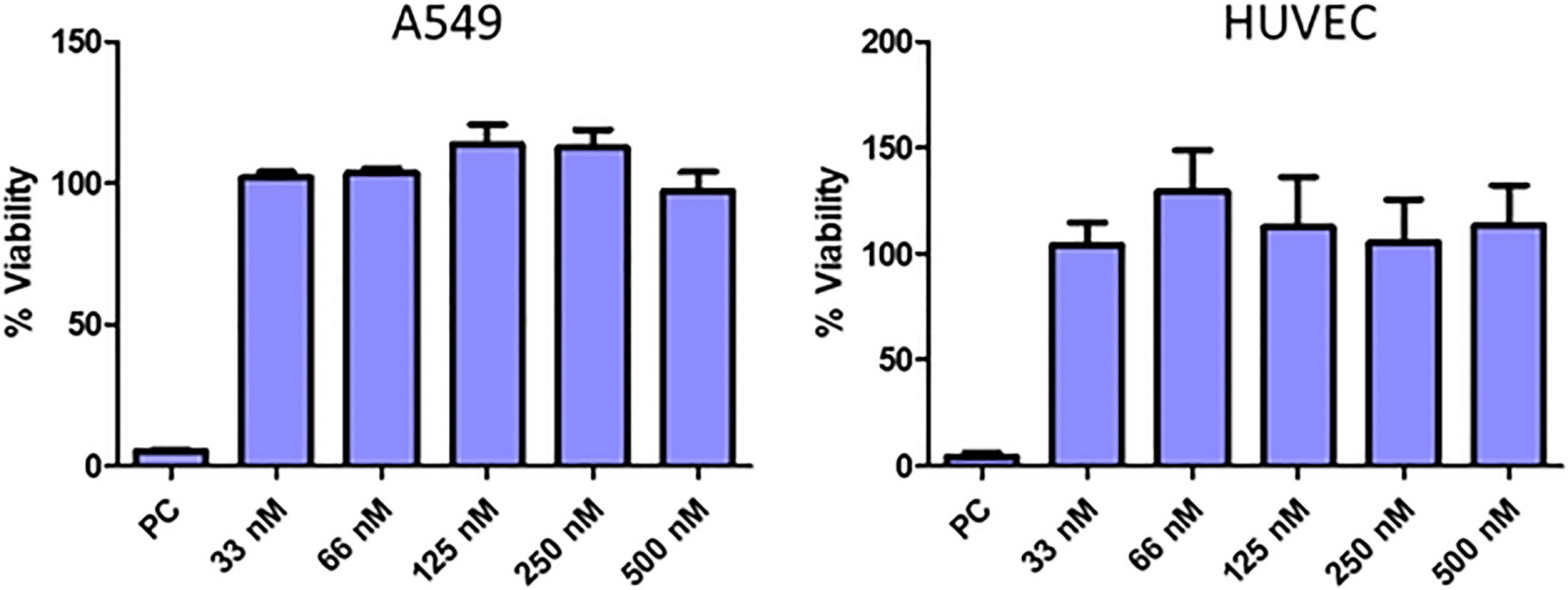
WST-1 assay analysis of cell viability following cNLC-TFD nanocomplex administration in A549 and HUVEC cells. Viability was assessed as percentage change against untreated negative control cells including cyclosporine positive control samples (PC). (n = 3 ±SEM).

In anticipation of future testing in MRSA infected *in vivo* systems, mammalian cell viability levels were tested over a TFD dose range of 500 nM– 33 nM. Nanocomplexes toxicity was assessed using the A549 alveolar cell line and primary human vascular endothelial cells (HUVEC) at 24 hours post-administration (Fig 7, S10 Table). This allow analysis in both cell line and primary cell types as well as being reflective of cell types where local (pulmonary) and systemic (circulatory system) MRSA infections may occur.

On analysis of cNLC-TFD toxicity in HUVEC cells (Fig 7), it was found that there were very low levels of toxicity observed. Viability was close to or above 100% in all doses tested with cells treated using 500 nM TFD/252 μg cNLC concentration (four times above that needed for enhancing vancomycin antimicrobial activity) remaining at 97% viability. Similarly, A549 alveolar epithelial cells demonstrated no decreases in cell viability at all dose ranges tested (Fig 7). Finally, in all cell lines tested, cyclosporine positive controls were observed to be functioning normally with all cell lines demonstrating significant decreases in viability. These results also further emphasised that the cNLC-TFD nanocomplex doses used to enhance the antimicrobial effect were via TFD-mediated transcription inhibition and not simply functioning through a particle-mediated cytotoxic effect.

### Erythrocyte integrity following incubation with cNLC-TFD nanocomplexes

**Fig 8:**
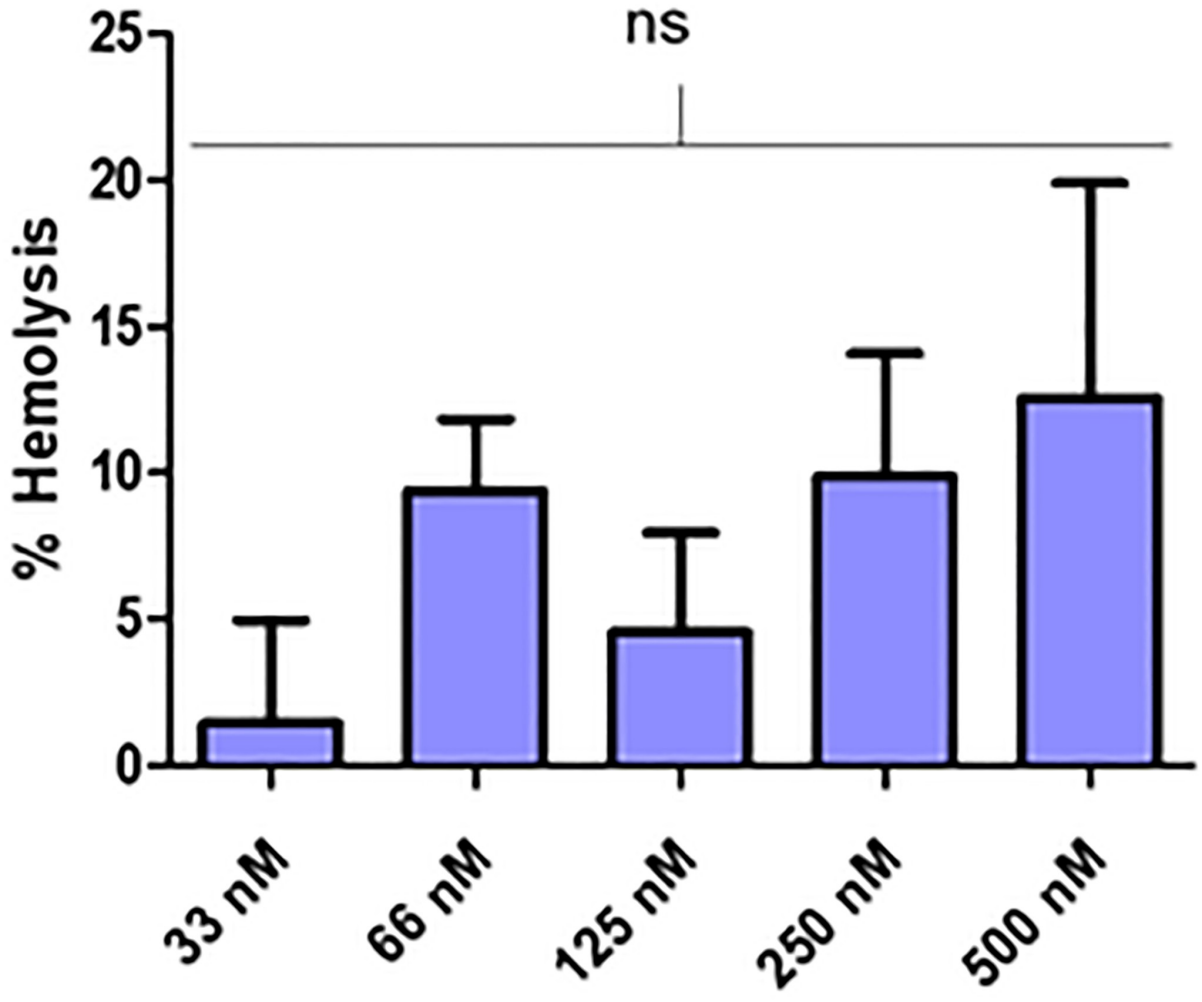
Percentage of haemolysis from whole human blood following incubation with cNLC-TFD nanocomplexes. Samples were incubated for 90 minutes at 37°C (n = 6 ±SEM, one-way ANOVA).

Due to the likelihood that any future *in vivo* administration of cNLC-TFD would occur through IV injection or perfusion, the potential for lysis of circulating erythrocytes was also assessed *in vitro*. Following 90 minutes of incubation and separation of the supernatant, it was found that cNLC-TFD nanocomplexes resulted in only low levels of haemolysis (Fig 8, S11 Table). The highest value recorded was 12.55% (±7.35%) haemolysis in the 500 nM TFD concentration sample, while haemolysis produced by lower concentrations of cNLC-TFD were below 10%. Furthermore, there was no statistically significant differences determined to be present between the dose ranges.

## Discussion

Overcoming resistance among pathogenic bacteria, especially MRSA, remains a priority for the development for the next generation of antimicrobial compounds. Unfortunately, mechanisms of drug resistance have been found to be highly adaptable and diverse in nature. These include target modifications (commonly through the development of mutations) that prevent binding of antimicrobials, production of enzymes that modify the antimicrobial to prevent drug binding, or decreasing the effective concentration of antimicrobials through efflux pumps to name but a few [1, 28].

Normally, the introduction of a novel antimicrobial molecule is followed immediately by the selection of antimicrobial resistant strains. Recently, this has already become apparent in the case of the small molecule drug bedaquiline. Approved for multi-drug-resistant tuberculosis in 2012, resistant variants are now being described [29–31]. Therefore, instead of entering into the typical cycle of new antimicrobial—resistance, our study attempted to address the issue at the genetic level, by using TFDs targeting essential transcription factors. This was with the rationale that, since the TFD DNA sequence being introduced exists normally in *S*. *aureus*, it will be much less likely to develop new resistance mechanisms to its presence. Furthermore, in our study, the specific TFD sequence binds the transcription factor WalR which is the regulator of the WalK/R two-component regulatory system in *S*. *aureus*. This protein is highly conserved and essential for the viability of this microorganism. WalR plays a key role in controlling cell wall metabolism and membrane composition, and it has been reported to be implicated in resistance to vancomycin [20, 21, 32]. TFDs as a gene inhibition strategy were especially attractive in prokaryotes given that, due to the cytoplasmic location of bacterial genomic DNA, they would only need to get inside the bacterial cells to exert an effect (rather than traverse the additional barrier of the nuclear membrane as in eukaryotes). While this benefit is counterbalanced somewhat by the presence of the bacterial cell wall, it has previously been demonstrated that it is possible fully internalize nucleic acid-nanoparticles into Gram-positive bacteria [33].

Considering this, to protect against degradation and aid in overcoming the bacterial cell wall and membrane permeability barriers, TFDs were complexed to two different nanocarriers. Both consisted of a nanoemulsion core surrounded either by a combination of PEG and cationic lipids (cNLCs) or a chitosan shell (CS-NCs). These were complexed with the TFD in two different fashions, with cNLCs being complexed separately with TFDs following nanoparticle formulation and CS-NCs combining with TFD during formulation. Following complexation, both were analysed for particle behavior and, in the case of the cNLC-TFDs, high levels of stability over 72 hrs was observed. cNLC-TFD sizes in PBS remained stable in PBS and TSB up to 72hrs with more variation seen in the samples incubated in mammalian cell culture media. This was further reflected on examination of the zeta potential of the cNLC-TFD nanocomplexes, the cationic nature of the two was evident in PBS further demonstrating that all negatively charged TFD was effectively condensed at N/P = 32. When tested in the more complex A549, HUVEC and TSB media, more erratic surface charges were recorded over time. All three exhibited negative surface charge from the start with TSB and A549 media increasing in zeta potential over 72 hrs and samples in HUVEC continuing to decrease in zeta potential. This is highly likely to be related to protein and sugar aggregation on the particles in all cases [34] with the differences between groups depending on the specific components of each media. For example, the presence of growth factors in HUVEC medium which are absent from the A549 and TSB.

Most importantly, following gel electrophoresis analysis, it was especially evident that cNLC-TFD nanocomplex integrity was maintained in all buffers tested. Specifically, when cNLC and TFDs were decomplexed in the presence of NaOH, TFD demonstrated similar bands to those of control TFD. This was in keeping with previous work by the group in developing highly stable nanoemulsion based nanoparticles [35–37] and indicated that cNLCs-TFD nanocomplexes were not destroyed in *in vitro* test conditions.

Since TFD oligonucleotides were added to CS-NCs prior to formulation being finalized, it was necessary to determine the optimal loading efficiency prior to stability analysis. This was in contrast to cNLCs where the TFD is added after formulation and complexation efficiency was considered near-total based on their cationic surface charge in PBS and signal quenching under UV following electrophoresis. Therefore, when CS-NC-TFD nanocomplexes were examined following formulation, it was quickly determined that a saturation point in TFD loading efficiency was reach when any more than 100 μg of TFD was loaded per formulation. When TFD loading was kept below this cut off, entrapment efficiencies were found to be as high as 44% with the balance of this deemed lost during production. When analyzed for physico-chemical properties, CS-NC-TFD demonstrated high levels of stability up to 24 hrs as can be expected when its inner core is based on the colloidal stability of a nanoemulsion/solid lipid nanoparticle [38, 39].

The decrease in size observed after 72h incubation of TFD-loaded nanocarriers in PBS, TSB and MHII, could be attributed to a destabilization effect of the components of the medium resulting in aggregation upon interaction with ions and proteins leading to a general decrease of mean hydrodynamic diameter. Similarly, the decrease in Z-potential value is in agreement with the high ionic strength of the medium. These results confirmed the interaction of medium components on nanocarrier surface which have been previously described as having dramatic impacts on particle size and surface charge [34].

Following initial characterization of both TFD nanocomplexes, these were first tested for antimicrobial activity against a methicillin susceptible strain of *S*. *aureus*. It was thought prudent to begin with a more treatable strain of *S*. *aureus* to establish the potency of the TFD-nanocarrier system in general. It was also thought logical to assess the activity of the TFD-nanocarriers alone and in the presence of vancomycin. This is since it is likely that any antimicrobial gene therapy treatments to be tested in patients will likely be administered in conjunction with the best available care (i.e. vancomycin). By use of the checkerboard assay, it was possible to rapidly assess the *in vitro* efficacy for both vancomycin and TFD-nanocarriers both alone and in tandem. From initial testing in MSSA, the cNLC-TFDs in the absence of vancomycin were not found to totally eliminate the bacteria. This made it impossible to determine a MIC using only the cNLC-TFDs at the concentrations tested. To date, TFDs have been successfully delivered to a range of mammalian cells (comprehensively reviewed by Ulasov *et al*.[40]) but remaining under-investigated in bacterial culture. At the time of writing, TFDs have been successfully delivered to *Escherichia coli* and *Clostridium difficile* using highly cationic carriers [17, 41, 42] but have yet to be established in *S*. *aureus* cultures.

However, on co-administration with vancomycin, it was found that the cNLC-TFDs exhibited a “boosting effect” and the MIC of vancomycin was decreased by 50% using TFD concentrations as low as 63 nM. While this could not be described as synergy at this point since the TFD did not exhibit antimicrobioal effect by itself, it was clear that the increase in efficacy was due to the cNLC-TFD reaching its site of action and consequently increasing vancomycin susceptibility. This was made clear when the increase in efficacy was not observed when a non-*S*. *aureus* specific TFD, cNLC alone or WalR-TFD non-encapsulated were used instead. Unfortunately, no antimicrobial effect was observed using the CS-NC-TFD nanocomplexes, either alone or in combination with vancomycin. This was surprising given the known suitability of chitosan particles as a delivery agent for antimicrobials against *S*. *aureus* [43]. Considering the anionic surface charge observed for the CS-NC-TFDs, it is likely that these nanocarriers were electrostatically repelled from the bacteria and were unable to exert an effect as has been previously demonstrated in mammalian cells [44, 45] and in MRSA [46]. Combined with the challenges posed by the rapid doubling time of bacteria, it is likely that any positive effects elicited by the few successful CS-NC-TFD transfections would be rapidly eclipsed by unaffected replicating bacteria. Therefore, further optimisation of this nanocarrier was deemed necessary before reapplying it to TFD delivery to *S*. *aureus* and it was not progressed any further in this study.

Having demonstrated a clear ability to enhance the efficacy of vancomycin in MSSA strains, the cNLC-TFDs were then progressed to antimicrobial activity experiments involving MRSA. We found that the MRSA strain was less susceptible (MIC 1.2 μg/mL) to vancomycin than the MSSA strain (MIC 0.6 μg/mL), and it was hypothesized that this finding represented a general decrease in susceptibility, and thus immediately represented a more challenging environment for TFD-mediated therapy.

On visual inspection, the ability of cNLC-TFDs to enhance the potency of vancomycin was not immediately clear as was the case with MSSA. However, absorbance analysis demonstrated that antimicrobial effect was very clearly retained while not entirely inhibiting growth of the bacteria. Specifically, sub-MIC (0.6 μg/ml) vancomycin and cNLC-TFD combination therapy resulted in significantly higher levels of bacterial growth inhibition compared to 0.6 μg/ml vancomycin treatment alone (54% vs 10% respectively). Furthermore, absorbance readings also indicated that cNLC-TFD administration alone was capable of up to a 32% reduction in MRSA viability, thus demonstrating a standalone ability of the cNLCs to promote transport of TFD molecule through the bacterial cell wall and membrane. This gave further weight to initial theories that the TFD was being successfully delivered and was biologically active but was insufficient to clear the bacteria on its own due to the logarithmic growth of the *S*. *aureus*. It was also noted that while the combined therapy was not significantly better than cNLC-TFD alone, cNLC-TFD administered on its own yields no significant improvement in antimicrobial effect compared to vancomycin. In comparison, the dual therapy approach is significantly better than vancomycin alone beyond the sum of its parts. It is now critical to recognize that in any clinical setting the rationale is not to add vancomycin to improve cNLC-TFD efficacy but rather the inverse. This is especially so given that any potential clinical trial will strive to continue best clinical care in conjunction with the test compound.

Therefore, while it was not possible to claim synergy in terms of relative MICs of vancomycin and cNLC-TFDs, it is possible to make this claim in terms of percentage rates of bacterial growth inhibition. Both sub-MIC vancomycin and cNLC-TFDs elicit an antimicrobial effect in MRSA and that the combination of the two results in higher levels of growth inhibition than the sum of their parts. This effect is thought to occur since both vancomycin and the TFDs inhibit *S*. *aureus* formation of the cell wall. The TFDs inhibit the WalR pathway as previously described, and vancomycin has long been known to inhibit the second stage of cell wall synthesis and may also affect permeability of the cell membrane [9, 10].

Finally, to ensure that administered cNLC-TFDs do not result in off-target effects or cytotoxic events, these were tested in relevant primary and cell line samples. All similar concentrations as used to produce anti-bacterial activity were found to result in no decrease in cell viability. Furthermore, haemolysis levels were minimal up to 500 nM concentrations of cNLC-TFD. All samples were roughly at 10% haemolysis and the preferred dose of 125 nM TFD was below 5% which was within the acceptable limits for nanoparticle-induced haemolysis [47, 48]. Thus, this study has developed a dual delivery approach that demonstrates a clear improvement over current best practice and is well tolerated by mammalian cells.

## Conclusions

Co-administration of nanomedicine-based products and free small molecule drugs remains an under-explored option but is a logical progression in the clinical development of nanomedicines. Considering that antimicrobial resistance is a persistent and constantly evolving issue in treating bacterial infection combined with the lack of novel small molecule drugs in the pipeline, serious consideration needs to be given to methods that increase the potency of currently available drugs. In this study, it was found that TFD molecules complexed with suitable nanocarriers are capable of significantly improving the potency of vancomycin against MSSA. When dual therapy was applied against MRSA cultures, a clear synergistic effect in decreasing bacterial growth was evident. This approach has remained virtually unreported but highlights a new possibility in overcoming resistance mechanisms in *S*. *aureus*. Optimizations of the nanocarrier composition and the sequence and structure of the TFD molecule are being carried out in order to further improve their combined efficacy against MRSA. Following this, it is hoped that in-depth screening in biofilm models and *in vivo* infection studies may occur.

## Supporting information

S1 Table Minimal data set of cNLC-TFD sizes (nm) at N/P = 32 over a 72-hour timeframe in a variety of biological buffers. (DOCX)

S2 Table Minimal data set of cNLC-TFD poly-dispersity indices at N/P = 32 over a 72-hour timeframe in a variety of biological buffers. (DOCX)

S3 Table Minimal data set of cNLC-TFD zeta potential (mV) at N/P = 32 over a 72-hour timeframe in a variety of biological buffers. (DOCX)

S4 Table Minimal data set of agarose gel electrophoresis to quantify the TFD entrapped in chitosan nanocapsules using fluorescence intensity (a.u.). (DOCX)

S5 Table Minimal data set of drug loading and entrapment efficiency of TFD-CS-NCs. (DOCX)

S6 Table Minimal data set of size analysis (nm) of TFD-CS-NC nanocarriers over a 72-hour timeframe in a variety of storage and biological buffers. (DOCX)

S7 Table Minimal data set of poly-dispersity analysis of TFD-CS-NC nanocarriers over a 72-hour timeframe in a variety of storage and biological buffers. (DOCX)

S8 Table Minimal data set of zeta potential analysis (mV) of TFD-CS-NC nanocarriers over a 72-hour timeframe in a variety of storage and biological buffers. (DOCX)

S9 Table Minimal data set of Synergy assay demonstrating enhanced antimicrobial effect against MRSA strain CECT 5190 with each row representing an independent experiment. (DOCX)

S10 Table Minimal data set of WST-1 assay analysis of cell viability following cNLC-TFD nanocomplex administration in A549 and HUVEC cells. Each row represents the averaged % viability from an independent experiment.
(DOCX)

S11 Table Minimal data set of percentage of haemolysis from whole human blood following incubation with cNLC-TFD nanocomplexes with haemolysis compared to untreated. Each row represents the averaged % haemolysis from an independent experiment (*outlier data).
(DOCX)

S1 Fig Synergy assay demonstrating enhanced bacterial killing of *S*. *aureus* strain CECT 794 following co-administration of free vancomycin (μg/ml) and cNLC-TFD nanocomplexes. A) 96 well plate layout and B) photograph of assay plate after addition of Alamar blue, revealing the enhanced vancomycin susceptibility of *S*. *aureus* in the presence of TFD at the point of the blue arrow (n = 4).
(TIF)

S2 Fig Reduced sample assay investigating bacterial killing of *S*. *aureus* strain CECT 794 following co-administration of free vancomycin (μg/ml) alone (rows A+B 1–5), combined with WalR-specifc TFD CS-NC-TFD nanocomplexes (rows C-F 1–5), combined with non-WalR specific/non-targeting TFD (rows A+B 7–11) and free non-WalR TFD (rows C-F 7–11). A) 96 well plate layout and B) photograph of assay plate after addition of Alamar blue, revealing no improvement in MIC using WalR TFD-CS-NCs at 125 nM (rows C+D 1–5) or 8 nM TFD (rows E+F 1–5) (n = 6).
(TIF)

S3 Fig Reduced sample assay investigating bacterial killing of *S*. *aureus* strain CECT 794 following co-administration of free vancomycin (μg/ml) and non-WalR specific cNLC-TFD nanocomplexes. A) 96 well plate layout and B) photograph of assay plate after addition of Alamar blue, revealing no improvement in MIC using WhiB7 TFD-cNLCs at 125 nM (rows C+D 1–5) (n = 6).
(TIF)
