# Supplementary material for: Co-delivery of free vancomycin and transcription factor decoy-nanostructured lipid carriers can enhance inhibition of methicillin resistant Staphylococcus aureus (MRSA)
Source: PLoS One. 2019 Sep 3;14(9):e0220684. doi: 10.1371/journal.pone.0220684 (PMC6719865; doi:10.1371/journal.pone.0220684)
Supplement: S1 Table — (DOCX) [file pone.0220684.s001.docx]

**S1 Table. Minimal data set of cNLC-TFD sizes (nm) at N/P=32 over a 72-hour timeframe in a variety of biological buffers.**

|  | **PBS** | | | **TSB** | | | **HUVEC Media** | | | **A549 Media** | | |
| --- | --- | --- | --- | --- | --- | --- | --- | --- | --- | --- | --- | --- |
| **T=0** | 40.64 | 39.94 | 39.28 | 49.98 | 42.58 | 40.98 | 262.2 | 282.7 | 301.1 | 126 | 124 | 128.1 |
| **T=24** | 39.01 | 39.14 | 40.11 | 44.69 | 43.89 | 43.02 | 365.1 | 411.2 | 450.9 | 49.69 | 48.84 | 47.85 |
| **T=72** | 41.85 | 41.13 | 40.34 | 46.08 | 45.44 | 45.51 | 116.2 | 117.4 | 122.9 | 39.68 | 39.29 | 39.43 |
