# Supplementary material for: Co-delivery of free vancomycin and transcription factor decoy-nanostructured lipid carriers can enhance inhibition of methicillin resistant Staphylococcus aureus (MRSA)
Source: PLoS One. 2019 Sep 3;14(9):e0220684. doi: 10.1371/journal.pone.0220684 (PMC6719865; doi:10.1371/journal.pone.0220684)
Supplement: S2 Table — (DOCX) [file pone.0220684.s002.docx]

**S2 Table. Minimal data set of cNLC-TFD poly-dispersity indices at N/P=32 over a 72-hour timeframe in a variety of biological buffers.**

|  | **PBS** | | | **TSB** | | | **HUVEC Media** | | | **A549 Media** | | |
| --- | --- | --- | --- | --- | --- | --- | --- | --- | --- | --- | --- | --- |
| **T=0** | 0.176 | 0.174 | 0.139 | 0.118 | 0.221 | 0.155 | 0.27 | 0.264 | 0.289 | 0.319 | 0.38 | 0.322 |
| **T=24** | 0.143 | 0.149 | 0.169 | 0.252 | 0.216 | 0.172 | 0.215 | 0.222 | 0.262 | 0.305 | 0.307 | 0.309 |
| **T=72** | 0.212 | 0.216 | 0.185 | 0.27 | 0.26 | 0.27 | 0.416 | 0.413 | 0.396 | 0.167 | 0.177 | 0.16 |
