# Supplementary material for: Co-delivery of free vancomycin and transcription factor decoy-nanostructured lipid carriers can enhance inhibition of methicillin resistant Staphylococcus aureus (MRSA)
Source: PLoS One. 2019 Sep 3;14(9):e0220684. doi: 10.1371/journal.pone.0220684 (PMC6719865; doi:10.1371/journal.pone.0220684)
Supplement: S3 Table — (DOCX) [file pone.0220684.s003.docx]

**S3 Table. Minimal data set of cNLC-TFD zeta potential (mV) at N/P=32 over a 72-hour timeframe in a variety of biological buffers.**

|  | **PBS** | | | | **TSB** | | | | **HUVEC Media** | | | | **A549 Media** | | | |
| --- | --- | --- | --- | --- | --- | --- | --- | --- | --- | --- | --- | --- | --- | --- | --- | --- |
| **T=0** | 4.43 | 3.02 | -1.29 |  | -1.22 | -3.3 | -4.35 | -4.78 | -2.84 | -3.58 | -4.46 |  | -4.86 | -3.17 | -3.87 | -4.73 |
| **T=24** | 1.61 | 5.41 | 6.43 | 5.78 | -3.19 | -4.66 | -6.04 | -6.56 | -4.32 | -5.93 | -8.02 |  | -5.62 | -11.4 | -12.3 | -11.5 |
| **T=72** | 1.67 | 3.33 | 3.27 | 2.99 | -0.15 | -0.124 | -0.224 |  | -9.03 | -10.4 | -8.3 |  | -2.15 | -2.22 | -3.63 | -2.73 |
