# Supplementary material for: Co-delivery of free vancomycin and transcription factor decoy-nanostructured lipid carriers can enhance inhibition of methicillin resistant Staphylococcus aureus (MRSA)
Source: PLoS One. 2019 Sep 3;14(9):e0220684. doi: 10.1371/journal.pone.0220684 (PMC6719865; doi:10.1371/journal.pone.0220684)
Supplement: S4 Table — (DOCX) [file pone.0220684.s004.docx]

**S4 Table. Minimal data set of agarose gel electrophoresis to quantify the TFD entrapped in chitosan nanocapsules using fluorescence intensity (a.u.)**

| **Lane** | **A** | **B** | **C** |
| --- | --- | --- | --- |
| 1 | 0 | 8.136.648 | 0 |
| 2 | 7.829.891 | 6.944.770 | 11.056.962 |
| 3 | 5.891.406 | 3.307.770 | 8.861.841 |
| 4 | 4.099.234 | 1.632.527 | 5.328.305 |
| 5 | 2.154.941 | 0 | 2.717.305 |
| 6 | 913.527 | 0 | 1.111.113 |
| 7 | 419.698 | 4.403.598 | 930.991 |
| 8 | - | 5.077.598 | 3.538.548 |
| 9 | 3.209.941 | 5.224.598 | 2.745.134 |
| 10 | 2.945.477 | 4.217.305 | 2.800.305 |
| 11 | 3.603.527 | 3.351.891 | 1.813.841 |
| 12 | 3.560.648 | 4.014.891 | 0 |
| 13 | 0 | 0 |  |
