# Supplementary material for: Co-delivery of free vancomycin and transcription factor decoy-nanostructured lipid carriers can enhance inhibition of methicillin resistant Staphylococcus aureus (MRSA)
Source: PLoS One. 2019 Sep 3;14(9):e0220684. doi: 10.1371/journal.pone.0220684 (PMC6719865; doi:10.1371/journal.pone.0220684)
Supplement: S5 Table — (DOCX) [file pone.0220684.s005.docx]

**S5 Table. Minimal data set of drug loading and entrapment efficiency of TFD-CS-NCs**

| **TFD initially added (μg)** | **50** | **100** | | **200** |
| --- | --- | --- | --- | --- |
| **Repetition number** | I | I | II | I |
| **Drug loading (DL) %** | 0.068 | 0.110 | 0.157 | 0.246 |
|  | 0.062 | 0.115 | 0.138 | 0.191 |
|  | 0.076 | 0.128 | 0.182 | 0.195 |
|  | 0.075 |  |  | 0.126 |
| **Entrapment efficiency (EE) %** | 41 | 35 | 50 | 32 |
|  | 37 | 37 | 44 | 24 |
|  | 45 | 41 | 58 | 25 |
|  | 45 |  |  | 16 |
