# Supplementary material for: Co-delivery of free vancomycin and transcription factor decoy-nanostructured lipid carriers can enhance inhibition of methicillin resistant Staphylococcus aureus (MRSA)
Source: PLoS One. 2019 Sep 3;14(9):e0220684. doi: 10.1371/journal.pone.0220684 (PMC6719865; doi:10.1371/journal.pone.0220684)
Supplement: S6 Table — (DOCX) [file pone.0220684.s006.docx]

**S6 Table. Minimal data set of size analysis (nm) of TFD-CS-NC nanocarriers over a 72-hour timeframe in a variety of storage and biological buffers.**

|  | **0 h** | **24 h** | **72 h** |
| --- | --- | --- | --- |
| **H2O** | 372.5 | 331.4 | 369.4 |
| **PBS** | 375.2 | 323.2 | 246.9 |
| **MHII** | 398.5 | 347.3 | 248.6 |
| **TSB** | 375.1 | 344.3 | 174.7 |
