# Supplementary material for: Co-delivery of free vancomycin and transcription factor decoy-nanostructured lipid carriers can enhance inhibition of methicillin resistant Staphylococcus aureus (MRSA)
Source: PLoS One. 2019 Sep 3;14(9):e0220684. doi: 10.1371/journal.pone.0220684 (PMC6719865; doi:10.1371/journal.pone.0220684)
Supplement: S7 Table — (DOCX) [file pone.0220684.s007.docx]

**S7 Table. Minimal data set of poly-dispersity analysis of TFD-CS-NC nanocarriers over a 72-hour timeframe in a variety of storage and biological buffers.**

|  | **0 h** | | | | | | | | | |
| --- | --- | --- | --- | --- | --- | --- | --- | --- | --- | --- |
| **H2O** | 0.314 | 0.286 | 0.264 | 0.316 | 0.224 |  |  |  |  |  |
| **PBS** | 0.315 | 0.359 | 0.212 | 0.302 | 0.332 |  |  |  |  |  |
| **MHII** | 0.286 | 0.333 | 0.338 | 0.223 | 0.333 | 0.295 | 0.22 |  |  |  |
| **TSB** | 0.282 | 0.323 | 0.176 | 0.263 | 0.255 | 0.196 | 0.32 | 0.232 | 0.235 | 0.315 |

|  | **24 h** | | | | | | | | | |
| --- | --- | --- | --- | --- | --- | --- | --- | --- | --- | --- |
| **H2O** | 0.112 | 0.206 | 0.255 | 0.192 | 0.275 | 0.199 | 0.217 | 0.217 | 0.219 | 0.277 |
| **PBS** | 0.241 | 0.223 | 0.212 | 0.242 | 0.101 |  |  |  |  |  |
| **MHII** | 0.144 | 0.155 | 0.287 | 0.268 | 0.111 | 0.133 |  |  |  |  |
| **TSB** | 0.083 | 0.103 | 0.04 | 0.042 | 0.061 | 0.039 | 0.026 | 0.021 | 0.071 | 0.044 |

|  | **72 h** | | | | | | | |
| --- | --- | --- | --- | --- | --- | --- | --- | --- |
| **H2O** | 0.31 | 0.326 | 0.276 | 0.322 |  |  |  |  |
| **PBS** | 0.167 | 0.205 | 0.182 | 0.163 | 0.113 | 0.193 | 0.148 | 0.124 |
| **MHII** | 0.052 | 0.07 | 0.074 | 0.078 | 0.094 | 0.058 | 0.04 |  |
| **TSB** | 0.136 | 0.266 | 0.139 | 0.148 | 0.265 | 0.222 |  |  |
