# Supplementary material for: Co-delivery of free vancomycin and transcription factor decoy-nanostructured lipid carriers can enhance inhibition of methicillin resistant Staphylococcus aureus (MRSA)
Source: PLoS One. 2019 Sep 3;14(9):e0220684. doi: 10.1371/journal.pone.0220684 (PMC6719865; doi:10.1371/journal.pone.0220684)
Supplement: S8 Table — (DOCX) [file pone.0220684.s008.docx]

**S8 Table. Minimal data set of zeta potential analysis (mV) of TFD-CS-NC nanocarriers over a 72-hour timeframe in a variety of storage and biological buffers.**

|  | **0 h** | | | | |
| --- | --- | --- | --- | --- | --- |
| **H2O** | -9.67 | -9.51 | -13.15 | -10.21 | -13.81 |
| **PBS** | -57.92 | -58.11 | -55.26 | -50.47 |  |
| **MHII** | -41.61 | -40.49 | -49.08 | -43.52 | -47.33 |
| **TSB** | -49.58 | -50.97 | -50.72 | -53.93 | -54.78 |

|  | **24 h** | | | | |
| --- | --- | --- | --- | --- | --- |
| **H2O** | -14.7 | -12.98 | -8.74 | -6.64 | -7.29 |
| **PBS** | -60.58 | -58.68 | -54.54 | -55.69 |  |
| **MHII** | -38.53 | -40.17 | -35.62 | -46.35 | -44.83 |
| **TSB** | -39.54 | -40.09 | -35.92 | -37.63 | -37.07 |

|  | **72 h** | | | | |
| --- | --- | --- | --- | --- | --- |
| **H2O** | -14.69 | -10.93 | -14.08 | -11.85 | -11.61 |
| **PBS** | -59.66 | -60.55 | -56.07 | -56.34 |  |
| **MHII** | -40.16 | -36.97 | -42.74 | -40.89 | -40.83 |
| **TSB** | -35.09 | -35.51 | -34.91 | -36.79 |  |
