# Supplementary material for: Co-delivery of free vancomycin and transcription factor decoy-nanostructured lipid carriers can enhance inhibition of methicillin resistant Staphylococcus aureus (MRSA)
Source: PLoS One. 2019 Sep 3;14(9):e0220684. doi: 10.1371/journal.pone.0220684 (PMC6719865; doi:10.1371/journal.pone.0220684)
Supplement: S9 Table — (DOCX) [file pone.0220684.s009.docx]

**S9 Table. Minimal data set of Synergy assay demonstrating enhanced antimicrobial effect against MRSA strain CECT 5190** **with each row representing an independent experiment.**

| **% viability** | **Untreated** | **0.6 μg/ml Vancomycin** | **cNLC-125 nM TFD** | **Vancomycin**  **+ cNLC-125 nM TFD** |
| --- | --- | --- | --- | --- |
|  | 100.000000 | 93.969600 | 87.236470 | 57.107230 |
|  | 100.000000 | 85.848100 | 72.820160 | 49.965950 |
|  | 100.000000 | 92.380630 | 44.917640 | 31.142900 |
