# Supplementary material for: Co-delivery of free vancomycin and transcription factor decoy-nanostructured lipid carriers can enhance inhibition of methicillin resistant Staphylococcus aureus (MRSA)
Source: PLoS One. 2019 Sep 3;14(9):e0220684. doi: 10.1371/journal.pone.0220684 (PMC6719865; doi:10.1371/journal.pone.0220684)
Supplement: S10 Table — Each row represents the averaged % viability from an independent experiment. (DOCX) [file pone.0220684.s010.docx]

**S10 Table. Minimal data set of WST-1 assay analysis of cell viability following cNLC-TFD nanocomplex administration in A549 and HUVEC cells. Each row represents the averaged % viability from an independent experiment.**

| **A549 % viability** | **PC** | **33 nM** | **66 nM** | **125 nM** | **250 nM** | **500 nM** |
| --- | --- | --- | --- | --- | --- | --- |
|  | 6.390603 | 100.000000 | 106.298500 | 109.227600 | 106.309900 | 103.272300 |
|  | 4.154647 | 100.000000 | 104.494100 | 104.536600 | 106.318400 | 104.513900 |
|  | 5.126509 | 105.976500 | 100.000000 | 127.573500 | 125.190800 | 84.021180 |

| **HUVEC % viability** | **PC** | **33 nM** | **66 nM** | **125 nM** | **250 nM** | **500 nM** |
| --- | --- | --- | --- | --- | --- | --- |
|  | 0.2351152 | 94.822100 | 88.188770 | 55.512490 | 56.362020 | 93.285970 |
|  | 7.657237 | 135.816300 | 166.010900 | 131.928500 | 91.071940 | 105.715100 |
|  | 1.296073 | 90.865100 | 105.313800 | 96.658060 | 125.841500 | 85.898080 |
|  | 8.063931 | 95.350530 | 158.696000 | 165.920800 | 148.412600 | 168.939300 |
