# Supplementary material for: Co-delivery of free vancomycin and transcription factor decoy-nanostructured lipid carriers can enhance inhibition of methicillin resistant Staphylococcus aureus (MRSA)
Source: PLoS One. 2019 Sep 3;14(9):e0220684. doi: 10.1371/journal.pone.0220684 (PMC6719865; doi:10.1371/journal.pone.0220684)
Supplement: S11 Table — Each row represents the averaged % haemolysis from an independent experiment (*outlier data). (DOCX) [file pone.0220684.s011.docx]

**S11 Table. Minimal data set of percentage of haemolysis from whole human blood following incubation with cNLC-TFD nanocomplexes with haemolysis compared to untreated.** Each row represents the averaged % haemolysis from an independent experiment (*outlier data)

| **33 nM** | **66 nM** | **125 nM** | **250 nM** | **500 nM** |
| --- | --- | --- | --- | --- |
| -1.047118 | 14.409290 | 6.146144 | 11.541090 | 29.319370 |
| 2.332039 | 7.477333 | 6.089211 | 17.508800 | 10.235050 |
| -7.803066 | 3.506736 | -11.658150 | -5.712958 | -14.746860 |
| 14.606190 | *23.140700** | 13.045600 | 23.921000 | 16.581320 |
| 7.603184 | *29.278300** | 7.144578 | 6.299784 | 0.5792917 |
| -7.057057 | 12.137140 | 6.559173 | 5.686771 | 33.361000 |
