# Supplementary figures and images for: Co-delivery of free vancomycin and transcription factor decoy-nanostructured lipid carriers can enhance inhibition of methicillin resistant Staphylococcus aureus (MRSA)
Source: PLoS One. 2019 Sep 3;14(9):e0220684. doi: 10.1371/journal.pone.0220684 (PMC6719865; doi:10.1371/journal.pone.0220684)

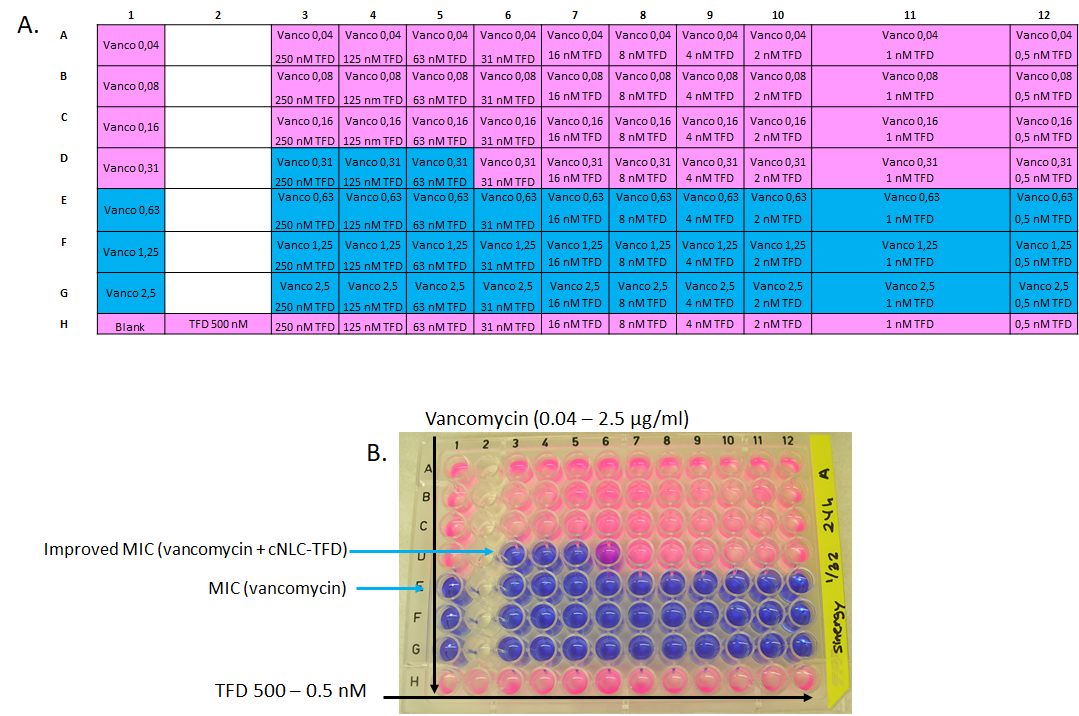

Supplement: S1 Fig — A) 96 well plate layout and B) photograph of assay plate after addition of Alamar blue, revealing the enhanced vancomycin susceptibility of S. aureus in the presence of TFD at the point of the blue arrow (n = 4). (TIF) [file pone.0220684.s012.tif]

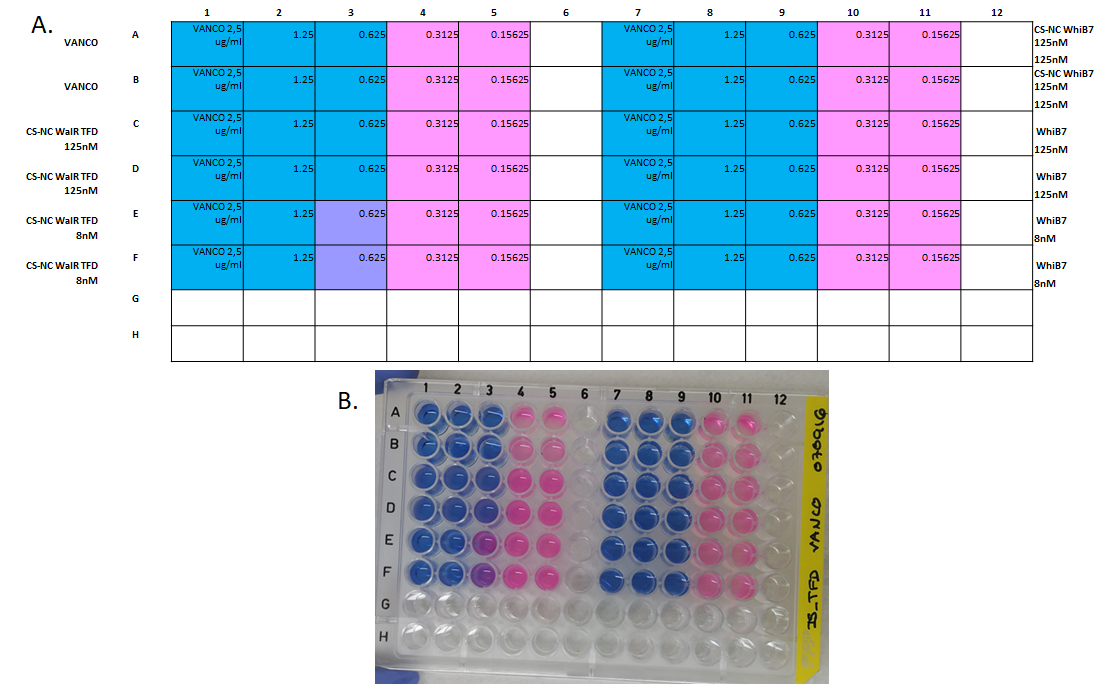

Supplement: S2 Fig — A) 96 well plate layout and B) photograph of assay plate after addition of Alamar blue, revealing no improvement in MIC using WalR TFD-CS-NCs at 125 nM (rows C+D 1–5) or 8 nM TFD (rows E+F 1–5) (n = 6). (TIF) [file pone.0220684.s013.tif]

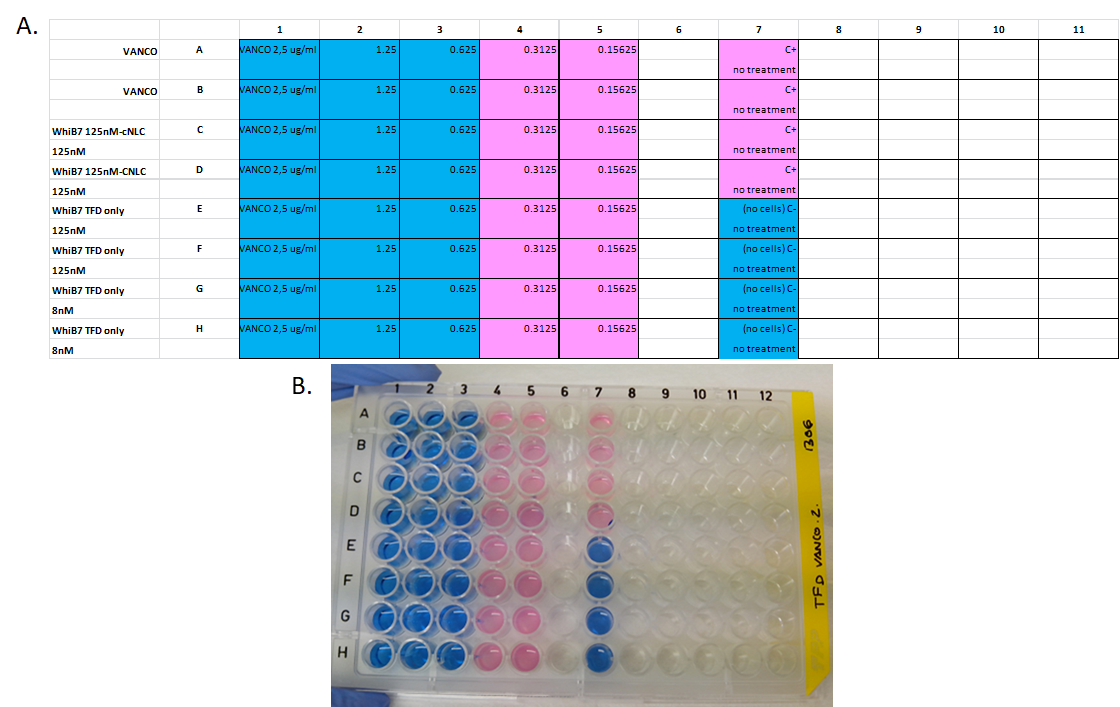

Supplement: S3 Fig — A) 96 well plate layout and B) photograph of assay plate after addition of Alamar blue, revealing no improvement in MIC using WhiB7 TFD-cNLCs at 125 nM (rows C+D 1–5) (n = 6). (TIF) [file pone.0220684.s014.tif]
